# Effects of eHealth Interventions on 24-Hour Movement Behaviors Among Preschoolers: Systematic Review and Meta-Analysis

**DOI:** 10.2196/52905

**Published:** 2024-02-21

**Authors:** Shan Jiang, Johan Y Y Ng, Kar Hau Chong, Bo Peng, Amy S Ha

**Affiliations:** 1 Department of Sports Science and Physical Education The Chinese University of Hong Kong Hong Kong China (Hong Kong); 2 School of Health and Society and Early Start Faculty of the Arts, Social Sciences and Humanities University of Wollongong Wollongong Australia

**Keywords:** preschooler, movement behaviors, eHealth, physical activity, sedentary behavior, sleep, mobile phone, review, systematic review

## Abstract

**Background:**

The high prevalence of unhealthy movement behaviors among young children remains a global public health issue. eHealth is considered a cost-effective approach that holds great promise for enhancing health and related behaviors. However, previous research on eHealth interventions aimed at promoting behavior change has primarily focused on adolescents and adults, leaving a limited body of evidence specifically pertaining to preschoolers.

**Objective:**

This review aims to examine the effectiveness of eHealth interventions in promoting 24-hour movement behaviors, specifically focusing on improving physical activity (PA) and sleep duration and reducing sedentary behavior among preschoolers. In addition, we assessed the moderating effects of various study characteristics on intervention effectiveness.

**Methods:**

We searched 6 electronic databases (PubMed, Ovid, SPORTDiscus, Scopus, Web of Science, and Cochrane Central Register of Controlled Trials) for experimental studies with a randomization procedure that examined the effectiveness of eHealth interventions on 24-hour movement behaviors among preschoolers aged 2 to 6 years in February 2023. The study outcomes included PA, sleep duration, and sedentary time. A meta-analysis was conducted to assess the pooled effect using a random-effects model, and subgroup analyses were conducted to explore the potential effects of moderating factors such as intervention duration, intervention type, and risk of bias (ROB). The included studies underwent a rigorous ROB assessment using the Cochrane ROB tool. Moreover, the certainty of evidence was evaluated using the GRADE (Grading of Recommendations Assessment, Development, and Evaluation) assessment.

**Results:**

Of the 7191 identified records, 19 (0.26%) were included in the systematic review. The meta-analysis comprised a sample of 2971 preschoolers, which was derived from 13 included studies. Compared with the control group, eHealth interventions significantly increased moderate to vigorous PA (Hedges *g*=0.16, 95% CI 0.03-0.30; *P*=.02) and total PA (Hedges *g*=0.37, 95% CI 0.02-0.72; *P*=.04). In addition, eHealth interventions significantly reduced sedentary time (Hedges *g*=−0.15, 95% CI −0.27 to −0.02; *P*=.02) and increased sleep duration (Hedges *g*=0.47, 95% CI 0.18-0.75; *P*=.002) immediately after the intervention. However, no significant moderating effects were observed for any of the variables assessed (*P*>.05). The quality of evidence was rated as “moderate” for moderate to vigorous intensity PA and sedentary time outcomes and “low” for sleep outcomes.

**Conclusions:**

eHealth interventions may be a promising strategy to increase PA, improve sleep, and reduce sedentary time among preschoolers. To effectively promote healthy behaviors in early childhood, it is imperative for future studies to prioritize the development of rigorous comparative trials with larger sample sizes. In addition, researchers should thoroughly examine the effects of potential moderators. There is also a pressing need to comprehensively explore the long-term effects resulting from these interventions.

**Trial Registration:**

PROSPERO CRD42022365003; http://tinyurl.com/3nnfdwh3

## Introduction

### Background

Physical activity (PA), sedentary behavior (SB), and sleep are integrated as “24-hour movement behaviors” owing to the collective effect on daily movement patterns. The 24-hour movement paradigm acknowledges the possibility of categorizing these behaviors according to their intensity levels across a full day. This encompasses a diverse range of activities, including sleep; SB (eg, screen time, reclining, or lying down); and light, moderate, or vigorous PA [1]. Globally, the “24-hour movement behaviors” paradigm has already been recognized and adopted into movement guidelines [2]. In 2020, the World Health Organization (WHO) released guidelines on PA and SB that incorporate all 3 movement behaviors [3]. The health benefits of engaging in PA, getting the recommended sleep, and reducing sedentary time are well documented. Recent reviews have shown a positive association between PA; sleep; and a wide range of child outcomes related to mental health, cognition, and cardiometabolism [4-6]. In addition, it is worth mentioning that different domains of SB can have varying health effects. For instance, non–screen-based sedentary activities such as reading or studying have been associated with favorable cognitive development in children [7]. Conversely, screen-based sedentary time, also referred to as “screen time,” has been found to have adverse effects on health-related outcomes [8]. Moreover, prior research has indicated that imbalances in 24-hour movement behaviors—specifically, elevated sedentary screen time coupled with diminished levels of PA and sleep—could potentially increase the risk of depression [9] and result in poor health-related quality of life [10]. Therefore, any change in one of the movement behaviors may lead to a compensatory increase or decrease in one or both behaviors.

However, insufficient healthy levels of 24-hour movement behaviors in early childhood have remained one of the most critical global public health challenges [11,12]. According to the WHO guidelines [3], preschool children are recommended to engage in adequate daily PA, consisting of 180 minutes, with 60 minutes dedicated to moderate to vigorous PA (MVPA). In addition, they should ensure sufficient sleep, ranging from 10 to 13 hours, while limiting sedentary recreational screen time to no more than 60 minutes per day. Unfortunately, a significant proportion of preschoolers do not meet the PA guidelines (<50% across studies) [13]. Furthermore, previous studies have consistently demonstrated that preschoolers exceed the screen time recommendations set by the WHO. A comprehensive meta-analysis of 44 studies revealed that only 35.6% of children aged between 2 and 5 years met the guideline of limiting daily screen time to 1 hour. Moreover, when examining the integration of 24-hour movement behaviors [8], another meta-analysis discovered that only 13% of children worldwide adhere to all 3 behavior guidelines [14].

Preschoolers play a crucial role in laying the foundation for long-term physical health and overall well-being [15,16]. Improving PA levels, minimizing SB, and prioritizing quality sleep in young children have multiple benefits, including positively influencing their physical fitness [17,18], promoting the development of motor and cognitive skills [19,20], and preventing childhood obesity [21] and associated health issues [14,22,23]. Several studies have shown that these healthy behavior patterns can shape lifelong habits that extend from childhood through adolescence and into adulthood [5,24].

Although these statistics are concerning, attempts to address the issue through various interventions have yielded inconsistent findings [25-28]. For instance, a meta-analysis of PA intervention studies involving preschoolers revealed only small to moderate effects in enhancing PA, suggesting room for improvement in achieving the desired outcomes [29]. In a meta-analysis conducted by Fangupo et al [30], no intervention effect was observed on daytime sleep duration for young children. Interestingly, earlier research has also elucidated overflow effects stemming from interventions focusing on a specific behavior, impacting other behaviors that were not the primary target. A systematic review highlighted that interventions aimed at enhancing PA in children aged <5 years led to a reduction in screen time by approximately 32 minutes [31]. It is crucial to understand that as time is finite, the durations dedicated to PA, sedentary time, and sleep are interconnected within 24 hours. Thus, we need effective interventions for preschool children that holistically address all components of 24-hour movement behaviors.

eHealth broadly refers to a diverse array of information and communication technologies used to facilitate the delivery of health care [32,33]. The rapid evolution of digitalization in recent decades has led to the widespread adoption of eHealth in interventions [28,34]. Recent reviews [35-38] suggest that with the global proliferation of eHealth interventions, health promotion via these platforms is evolving to become more accessible and user-friendly, garnering acceptance among adolescents and adults. Previous reviews have underscored the effectiveness of these digital platforms in enhancing various movement behavior outcomes across diverse age groups, including children aged 6 to 12 years [39], adolescents [40], adults [41], and older adults [42]. Specifically, a meta-analysis indicated that eHealth interventions have successfully promoted PA among individuals with noncommunicable diseases [43]. Another review showed that computer, mobile, and wearable technologies have the potential to mitigate sedentary time effectively [41]. Previous studies have targeted different participant groups to investigate the impact of eHealth on sleep outcomes. Deng et al [44] conducted a meta-analysis demonstrating that eHealth interventions for adults with insomnia are effective in improving sleep and can be considered a promising treatment. Nevertheless, a review focusing on healthy adolescents found that there has not been any school-based eHealth interventions focusing on sleep outcomes [45].

Indeed, child-centered strategies such as gamification are used in some digital apps and have been shown to encourage children’s PA [46-48]. A considerable body of work has addressed the pivotal role of parental influence and role modeling in cultivating healthy lifestyle habits in children [49,50]. Physical literacy, a multidimensional concept encompassing various aspects of PA such as the affective, physical, cognitive, and behavioral dimensions, plays a vital role in enhancing PA engagement [51]. Ha et al [52] conducted a web-based parent-focused intervention, revealing that enhancing parents’ physical literacy can effectively support children’s participation in PA. By understanding and promoting physical literacy, parents can provide valuable support to their children, fostering a lifelong commitment to healthy and active lifestyles. Although eHealth interventions offer promise, there are conflicting findings regarding their impact, especially when they are parent supported and targeted at young children. A previous meta-analysis examining eHealth interventions targeted at parents found no significant impact on children’s BMI. In addition, no studies have included children aged <5 years [50]. Similarly, a recent systematic review observed that eHealth interventions aimed at parents showed no significant effectiveness in enhancing PA levels in young children [53]. However, the prevalence of digital device use in young children has become widespread. For instance, studies conducted in England (the United Kingdom), Estonia, and the United States have reported that, on average, 83% of children aged 5 years use a digital device at least once a week [54]. Research also revealed that in the United States, approximately three-fourths of children had their own mobile device by the age of 4 years, and nearly all children (96.6%) used mobile devices [55]. Consequently, there is an urgent need to harness the potential of digital platforms and explore whether they can effectively deliver interventions to preschoolers [56].

### Objectives

In previous research, there has been a lack of studies examining the effectiveness of eHealth behavior change interventions among preschoolers. Although a systematic review found a significant effect of digital health interventions on the PA of preschoolers [53], this review did not include sedentary time and sleep in its inclusion criteria, and there is a lack of conclusive statements owing to the insufficient number of studies, and no quantitative methods were available for synthesizing the evidence on the effectiveness of eHealth interventions. To our knowledge, no systematic review or meta-analysis has distinctly investigated the effects of eHealth interventions on 24-hour movement behaviors in preschoolers or the factors that may influence their implementation. Therefore, the aims of this study were (1) to assess the effectiveness of eHealth interventions on 24-hour movement behaviors (improving PA and sleep duration and decreasing sedentary time) and (2) to examine the moderating effects of study characteristics (eg, intervention duration, intervention type, and outcome measurement tools) on intervention effectiveness.

## Methods

This review was registered with PROSPERO (CRD42022365003) and conducted in accordance with the PRISMA (Preferred Reporting Items for Systematic Reviews and Meta-Analyses) guidelines [57].

### Eligibility Criteria

This review included trials with a randomization procedure that examined the outcomes of interventions using information and communication technology. These interventions targeted at least 1 movement behavior in preschool children aged 2 to 6 years. Studies were excluded if (1) the control groups received intervention using eHealth technology and (2) published in a non-English language. Full details are provided in Multimedia Appendix 1 [58].

### Search and Selection

The following databases were systematically searched from inception to February 08, 2023: PubMed, Ovid, SPORTDiscus, Scopus, Web of Science, and Cochrane Central Register of Controlled Trials. We used the search terms “eHealth,” “Physical activity,” “Sedentary behavior,” “Sleep,” “preschooler,” and their Medical Subject Headings terms. The complete search strategy is described in Multimedia Appendix 2 [59-61]. A manual search of the reference lists of the included publications was performed to identify additional eligible studies for potential inclusion. Two independent reviewers (SJ and BP) screened the titles and abstracts and subsequent full-text articles for eligibility. Discrepancies that emerged during the selection process were effectively resolved through a discussion involving 3 authors (SJ, BP, and JYYN).

### Data Extraction

A comprehensive data extraction form was developed (SJ) and refined (SJ and BP) based on the Cochrane Handbook for Systematic Reviews of Interventions [62]. Extracted information included bibliographic details (authors, title, journal, and year); study details (country, design, retention rate); participants’ characteristics (number of children and demographics); intervention type (parent supported, teacher led, or child centered), intervention’s theoretical basis, duration, delivery tool, and intensity; comparison (sample size, activity type); outcomes (behavioral variables with baseline and postintervention means with SDs), and measurement tools. Regarding the categorization of intervention types, we have established a clear classification. Specifically, in child-centered interventions, children are the direct beneficiaries, participating autonomously with less guidance from guardians. This can be accomplished using an exergaming system or designed mobile health games. In parent-supported interventions, parents are involved in educational programs and instructions that improve parents’ knowledge of preschoolers’ healthy movement behaviors. A teacher-led intervention involves supervising preschoolers’ PA during school time or participating in structured PA sessions aimed at improving healthy indicators. For data that were either incomplete or absent within the main text, we sought to reach out to the respective authors through email correspondence.

### Risk of Bias

The included studies were assessed for risk of bias (ROB) using the revised Cochrane ROB2 tool [63]. The following domains of bias were assessed for each study: selection (random sequence generation and allocation concealment), performance and detection (masking of participants, personnel, and assessors), deviations from intended interventions, missing outcome data, measurement of the outcome, appropriateness of analysis (selection of the reported outcome), and bias arising from period and carryover effects (for crossover studies) [63]. The studies were ranked as low risk, some concerns, or high risk for each domain. The ROB was evaluated independently by 2 authors (SJ and BP). Any discrepancies were resolved through discussions with the author (JYYN).

### Outcomes and Data Synthesis

Our outcome targeted any of the following movement behaviors: PA (MVPA and total PA), sedentary time (screen time and sitting time), or sleep duration. Meta-analysis was conducted in R (version 4.2.1; R Group for Statistical Computing) using the *meta*, *metafor*, and *metareg* packages [64]. A random-effects model (Hartung-Knapp method) was used to calculate pooled estimates (Hedges *g*, a type of standardized mean difference) to account for variations in participants and measurement methods of movement behavior outcomes [65]. Multimedia Appendix 3 [63-65] describes the processing of missing data. Hedges *g* and their corresponding variances were calculated using the pre- and postintervention mean scores and SDs. However, if some studies had changes in baseline and postintervention data or if there were significant differences in their baseline data [59-61], we used the within-group difference in means and their SDs for intervention and control groups to calculate the effect size. Values of 0.2, 0.5, and 0.8 represent small, moderate, and large effect sizes, respectively. A positive effect size indicated a beneficial effect on the intervention group compared with the control group. The between-group heterogeneity of the synthesized effect sizes was examined using the Cochran Q test and *I*^2^ statistics. *I*^2^ values of 25%, 50%, and 75% indicated low, moderate, and high levels of heterogeneity, respectively. Subgroup analyses were conducted based on the following factors: (1) intervention duration (0-3 months vs >3 months) and (2) type of intervention (child centered, parent focused, or teacher led). (3) Types of outcome measurement tools (objective vs self-reported) and (4) ROB (low risk, some concerns, or high risks).

Furthermore, we performed meta-regression analyses to examine the impact of potential moderators on the overall effect size. Potential moderators included 5 variables, as specified in the subgroup analyses, and 2 continuous variables (sample size and intervention length). These variables were selected based on existing evidence that highlights their significant moderating effects on eHealth interventions targeting movement behaviors [53,66,67]. Sensitivity analyses were performed using the leave-one-out method. Publication bias was visualized using funnel plot symmetry and quantified using the Eggertest score, for which *P*<.05 indicates a significant publication bias [68].

### Quality Assessment of the Overall Evidence

GRADE (Grading of Recommendations, Assessment, Development, and Evaluation) 2 criteria were used to assess the certainty of evidence for the effect of eHealth interventions on the targeted outcomes [69,70]. The GRADE assessment was completed using GRADEpro, and the quality of evidence was classified as high (≥4 points overall), moderate (3 points), low (2 points), or very low (≤1 point) [70].

## Results

### Study Selection

**Figure 1:**
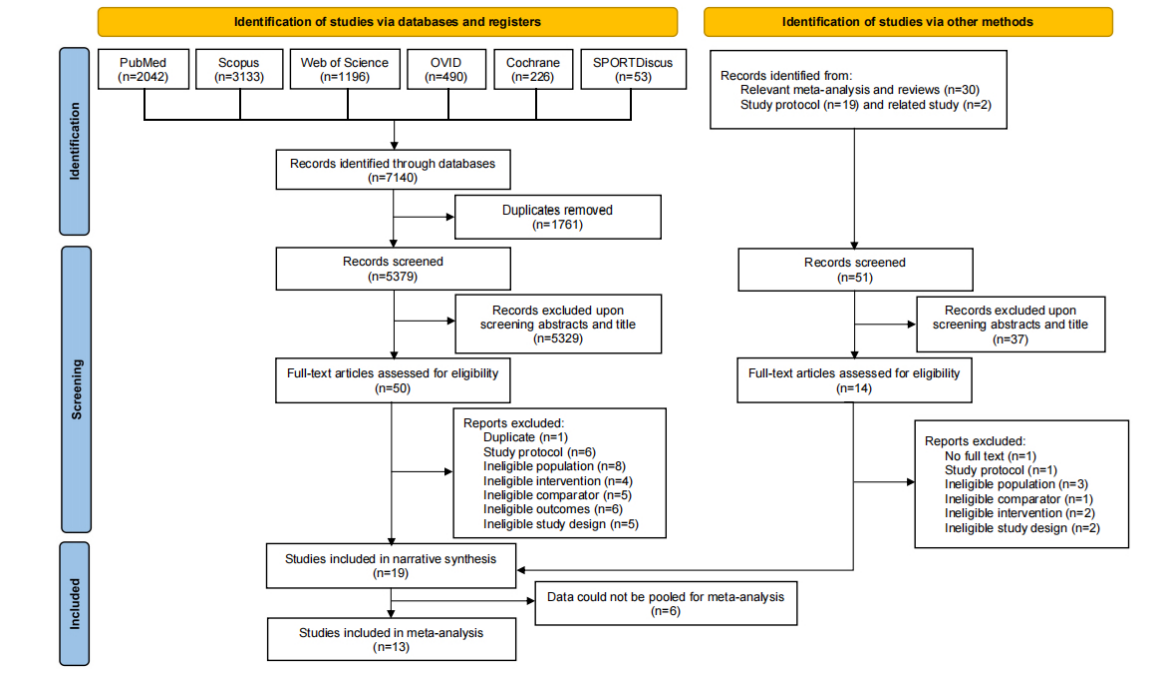
Flowchart of study selection.

The database search yielded 7140 records, with an additional 51 records identified from the reference lists of relevant systematic reviews. There were 64 articles screened for full text, and 45 articles were excluded. The reasons for exclusion are listed in Multimedia Appendix 4. A total of 19 studies reporting the effectiveness of interventions on movement behaviors were included in the systematic review [17,59-61,71-85], and 13 studies were included in the meta-analysis [59-61,76-85]. The PRISMA flowchart of the study selection process is shown in Figure 1 and PRISMA checklists are in Multimedia Appendices 5 and 6.

### Study Characteristics

The study characteristics are described in Table 1. In the 19 studies, 2971 preschoolers from 6 regions were included. A total of 18 studies were conducted in high-income countries, and only 1 study was conducted in an upper middle–income country, according to the World Bank classification (Multimedia Appendix 7) [86]. Most included studies were conducted during and after 2017. For the study design, 16 studies were 2-arm randomized controlled trials (RCTs), with 11 using a parallel group design [17,59-61,71-74,76,77,84], 2 being cluster RCTs [82,83], 2 pilot RCTs [79,81], and 1 crossover study [85]. The remaining 3 studies consisted of 2-arm experimental studies with a randomization procedure [75,78,80]. The sample size ranged from 34 preschoolers to 617 preschoolers. The study details are presented in Multimedia Appendices 8 and 9 [59-61,76-85].

**Table 1:** Characteristics of the included studies.

Study; country	Study design; participants’ age; sample size (I^a^ and C^b^); retention rate	Intervention type; delivery	Intervention components	Intervention duration, frequency	Control group	Targeted 24-h movement behavior outcomes
Andersen et al [82]; Norway	Two-arm cluster-randomized controlled trial; 3-4 y; 116 (I: 67 and C: 49); 89.66%	Teacher led; Facebook group	Premeetings (1 h) and follow-up meetings (1 h) in each ECEC^c^ institution, Start-up seminar (6 h) and 2 follow-up courses (3+3 h) with all the staff members, Ongoing planning and collective reflections practice in the ECEC institutions (weekly regular), Facebook group (4 mo) An equipment package.	4 mo	Maintain usual routines	PA^d^ and SB^e^
Delisle Nyström et al [60]; Sweden	Two-arm parallel randomized controlled trial; mean 4.5 y (SD 2 mo); 315 (I: 156 and C: 159); 83.49%	Parent supported; mHealth^f^ app	A smartphone app is available to parents for 6 mo, which contains 12 healthy behavior change–related themes, with a new theme being released every 2 wk (6 mo for parents involved into mHealth intervention with researchers’ support and instruction). There is no support, and parents are free to use the apps for the next 6 mo with follow-up.	6+6-mo follow-up without support	The control group received a basic pamphlet on dietary and PA behaviors.	PA and SB
Nyström et al [61]; Sweden	Two-arm parallel randomized controlled trial; mean 4.5 y (SD 2 mo); 315 (I: 156 and C: 159); 89.2%	Parent supported; mHealth app	A smartphone app is available to parents for 6 mo, which contains 12 healthy behavior change–related themes, with a new theme being released every 2 wk (6 mo for parents involved into mHealth intervention with researchers’ support and instruction). There is no support, and parents are free to use the apps for the next 6 mo with follow-up.	6 mo	The control group received a basic pamphlet on dietary and PA behaviors.	PA and SB
Haines et al [76]; United States	Two-arm randomized controlled trial; 2-5 y; 121 (I: 62 and C: 59); 94.21%	Parent supported; telephone call and texting	Motivational coaching by a health educator during 4 home visits and 4 health coaching telephone calls Mailed educational materials and incentives. Weekly texting on adoption of household routines	6 mo (monthly coaching calls and text messages twice weekly)	The control group received 4 monthly mailed packages that included educational materials.	SB and sleep
Ling et al [80]; United States	Two-group experimental study with randomization procedure; 3-5 y; 73 (I: 41 and C: 32); 94.52%	Parent supported; Facebook group	The caregiver Facebook-based program aimed to (a) offer health information, engage in family fun activities, and provide behavioral change strategies and tips. (b) it encouraged interactive positive communication among caregivers, fostering peer support. Caregivers were instructed to gradually increase their weekly step count by 500, with the goal of becoming positive role models for their preschoolers. Three face-to-face caregiver meetings were provided. The center-based preschooler program has been conducted. Preschoolers received 10-wk healthy eating and PAe participatory learning and fun games codelivered by teachers and interventionists 4 d per wk. (total 40 d) at their attended Head Start center. The session duration was 30 min per d. The child’s weekly letter to the caregiver was privately sent through Facebook Messenger. Caregivers were encouraged to engage in discussions with their preschooler about the letter over the weekend and share their thoughts and plans in response to the letter within the Facebook private group.	10 wk	Maintain usual routines	PA and SB
Downing et al [79]; Australia	Two-arm pilot randomized controlled trial; 2-4 y; 57 (I: 30 and C: 27); 92.98%	Parent supported; text message	A one-on-one discussion with each participant individually, either in person or over the phone, to set their goals for the program. Two goals were set around reducing their child’s SB, one is a screen time goal and another is an overall SB goal. Three personalized, interactive text messages (ie, the main mode of intervention delivery) per week were sent to parents for 6 wk (19 texts in total). The standard text messages included 2 behavioral messages with practical ideas and suggestions for limiting and displacing their child’s screen and sitting time, active play ideas, and monitoring and encouraging achievement of individual goals.	6 wk	Waitlist control	SB
Yoong et al [81]; Australia	Two-arm pilot randomized controlled trial; 3-6 y; 76 (I: 38 and C: 38); 48.68%	Parent supported; Web-based video, telephone call, and text messages	Web-based video: education prerecorded videos were sent to parents. The content targeted brief information about sleep benefits and targeted behaviors to implement sleep practices. Telephone call: approximately 2-4 wk after the video link was emailed, a trained psychologist provides parents support to implement the messages in the video. Text messages: 2 text messages were sent approximately 1 wk after the phone call. Text messages were tailored to parent’s engagement with the intervention and were aimed at encouraging parents to access the video and persist with implementing any new routines that had been put in place.	3 mo	Waitlist control	PA and sleep
Hoffman et al [83]; United States	Two-arm cluster-randomized controlled trial; 3-5 y; 58 (I: 27 and C: 31); 98.28%	Teacher led; study website	Teachers were trained by receiving an email about a WE PLAY website link, and the training was self-paced over a 4-wk period. Training activities were designed to be completed in five steps: (1) complete the web-based training, (2) view the video library, (3) read the game sheets and select games, (4) lead a structured and unstructured active play session and complete the teacher self-assessments, and (5) complete the supervisor support component (ie, observations, performance feedback, and teacher recognition)	4 wk	No other instructions related to PA	PA
Marsh et al [84]; New Zealand	Two-arm randomized controlled trial; 2-4 y; 54 (I: 27 and C: 27); 98.15%	Parent supported; study website	Parent-child dyads participated in a half-day, face-to-face workshop at the University of Auckland. The workshop is designed to introduce the program framework and provide a blueprint for parents to develop healthy lifestyle behaviors among children. After the workshop, all participants were given access to a study website, which provided all the workshop content, for a period of 6 wk.	6 wk	Waitlist control	SB and sleep
Marsh et al [84]; New Zealand	Two-arm randomized controlled trial; 2-4 y; 54 (I: 27 and C: 27); 98.15%	Parent supported; study website	Parent-child dyads participated in a half-day, face-to-face workshop at the University of Auckland. The workshop is designed to introduce the program framework and provide a blueprint for parents to develop healthy lifestyle behaviors among children. After the workshop, all participants were given access to a study website, which provided all the workshop content, for a period of 6 wk.	12 wk	Waitlist control	SB and sleep
Barkin et al [77]; United States	Two-arm randomized controlled trial; 3-5 y; 610 (I: 304 and C: 306); 75.74%	Parent supported; telephone call	A 12-wk intensive phase with weekly 90-min skills-building sessions via either in-person groups or telephone calls, A 9-mo maintenance phase with monthly coaching telephone calls. A 24-mo sustainability phase providing frequent cues to action (eg, texts, personalized letters, and monthly calls) to use parks and recreation programing for healthy family behaviors	12 mo (12-wk with 90 min per wk skills-building session via phone call and 9-mo weekly telephone call)	School-readiness program	PA and SB
Barkin et al [77]; United States	Two-arm randomized controlled trial; 3-5 y; 610 (I: 304 and C: 306); 75.74%	Parent supported; telephone call	A 12-wk intensive phase with weekly 90-min skills-building sessions via either in-person groups or telephone calls, A 9-mo maintenance phase with monthly coaching telephone calls. A 24-mo sustainability phase providing frequent cues to action (eg, texts, personalized letters, and monthly calls) to use parks and recreation programing for healthy family behaviors	24 mo	School-readiness program	PA and SB
Barkin et al [77]; United States	Two-arm randomized controlled trial; 3-5 y; 610 (I: 304 and C: 306); 75.74%	Parent supported; telephone call	A 12-wk intensive phase with weekly 90-min skills-building sessions via either in-person groups or telephone calls, A 9-mo maintenance phase with monthly coaching telephone calls. A 24-mo sustainability phase providing frequent cues to action (eg, texts, personalized letters, and monthly calls) to use parks and recreation programing for healthy family behaviors	36 mo	School-readiness program	PA and SB
Byun et al [78]; United States	Two parallel group experimental study with randomization procedure; 2-5 y; 115 (I: 57 and C: 58); 80.87%	Teacher led; wearable device monitor	One-week real-time PA monitoring system provided teachers with instant feedback on children’s PA levels. On the basis of the feedback, teachers were asked to self-regulate their classroom strategies to provide more PA opportunities as needed. Teachers were given the autonomy to design the intervention strategies with expectations for encouraging children to be more physically active.	1 wk	Maintain usual routines	PA and SB
Zeng et al [85]; United States	Two-arm randomized with 2-period crossover trial; 3-5 y; 34 (I: 18 and C: 16); 94.12%	Child centered; exergaming	Families in the exergaming group received a LeapTV educational video game console (LeapFrog Enterprises, Inc), with several age-appropriate exergames, including Sports!, Dance & Learn, Kart Racing Supercharged, Pet Play World, Dora and Friends, Ultimate Spider-Man, Bubble Guppies, Sofia the First, Paw Patrol, and Jake and the Never Land Pirates. Children used the active gaming system and engaged in exergaming 30 min per d for 5 d per wk.	12 wk, 30 min per session, 5 times per week	Maintain usual routines	PA and SB
Alexandrou et al [59]; Sweden	Two-arm parallel group individually randomized control trial; 2.5-3 y; 552 (I: 277 and C: 275); 91.12%	Parent supported; mHealth app	Participants in the intervention group were given immediate access to the MINISTOP^g^ 2.0 app (a mobile app). The intervention builds on the MINISTOP 1.0 digital platform and content over 6 mo. Parents used this app, receiving an extensive health-related information and feedback, to increase parental knowledge, skills, and self-efficacy to support and enable behavior change for improved diet and PA behaviors in children.	6 mo	Maintain usual routines	PA and SB
Garrison and Christakis [71]^g^; United States	Two-arm randomized controlled trial; 3-5 y; 617 (I: 303 and C: 314); 91.6%	Parent supported; a telephone call and DVD	Intervention sessions began with the initial home visit and was then followed by mailings and follow-up telephone calls with the case manager for 12 mo. The first 6 mailings also included DVDs with 5- to 10-min clips of suggested educational and prosocial shows. Case managers used their training to motivate parents to replace violent and age-inappropriate media content with content that was age appropriate and educational or prosocial in nature.	6 mo	A nutrition intervention, including monthly mailings, that encouraged families to decrease consumption of sugary drinks and increase fruit and vegetable intake.	Sleep
Sun et al [72]^g^; United States	Two-arm randomized controlled trial; 3-5 y; 32 (I: 16 and C: 16); 90.6%	Parent supported; tablet computer, videos, and telephone calls	The intervention consisted of 8 weekly 30-min, interactive, Cantonese, educational modules accessed on the web via tablet computers. The topics were as follows: (1) introduction to the 5-4-3-2-1-0 program;(2) energy balance—maintain a healthy weight; (3) what to feed my family—energy IN; (4) grocery shopping; (5) find fun in PA—energy OUT; (6) less sit, more fit—decrease screen time; (7) smart parenting; and (8) maintain a healthy weight for life. A total of 6 of the 8 lessons were in the format of a 10- to 15-min animated short video in Cantonese, and 2 lessons were in a talk show format hosted by a bicultural registered dietitian or health educator with Cantonese-speaking mothers of young children.	8 wk 30 min per week	Received weekly mailings of printed health information to preschool-aged children over the 8 wk	PA
Fu et al [73]^g^; United States	Two-arm randomized controlled trial; mean 4.9 (SD 0.7); 65 (I: 36 and C: 29); 98.5%	Child centered; exergaming	Exergaming was incorporated into the regular school routine. During the 12-wk exergaming intervention, children had one 30-min exergaming session per day for 5 d per wk, supervised by 1 trained research assistant and teachers. During the 30-min exergaming session, several active videogames were offered.	12 wk, 30 min per session for 5 times per week	Children in the control group had five 30-min active free-play sessions per week for 12 consecutive weeks, supervised by schoolteachers.	PA
Gao et al [17]^g^; United States	Two-arm randomized controlled trial; 4-6 y; 34 (I: 18 and C: 16); 94.12%	Child centered; exergaming	Parents were instructed to have their children perform a home-based educational exergaming using LeapTV gaming console for at least 30 min per session for 5 times per week beyond usual PA (duration: 12 wk) A phone call to parents 2 d after the LeapTV installation; and researchers also visited each family once or twice within the first 2 wk to encourage using LeapTV.	12 wk, 30 min per session for 5 times per week	Maintain regular PA patterns without any exergaming gameplay	PA
Trost et al [74]^g^; Australia	Two-arm randomized controlled trial; 3-6 y; 34 (I: 17 and C: 17); 94.12%	Child centered; mHealth app	Moovosity is a mobile app designed to promote the development of FMS^h^ and increase PA in young children. The game is started by touching the “LETS GO!” button, initiating a 10-min timer for the child and parent to actively play the game without the use of the app.	8 wk	Waitlist control	PA
Yarimkaya et al [75]^g^; Turkey	Two-group experimental study with randomization procedure; mean age 5.7; 42 (I: 21 and C: 21); 100%	Parent supported; WhatsApp	The parents were included in this private WhatsApp group. A 20- to 30-min PA session was held for 7 d per wk for 6 wk. Each PA session consisted of (1) a warm-up period of approximately 10 min, (2) a PA period of approximately 10 min, and (3) a cooling down period of approximately 10 min. Parents were sent 3 YouTube videos (approximately 10 min each) illustrating the 3 components of a 30-min session.	6 wk, 20- to 30-min PA session for 7 d per wk	Maintain usual routines	PA

^a^I: intervention.

^b^C: control.

^c^ECEC: early childhood education and care.

^d^PA: physical activity.

^e^SB: sedentary behavior.

^f^mHealth: mobile health.

^g^MINISTOP: mobile-based intervention intended to stop obesity in preschoolers.

^h^FMS: fundamental movement skills.

### Intervention Details

The included studies used various delivery channels of eHealth technologies for the intervention. Seven studies used smartphone apps [59-61,74] and social media (Facebook and WhatsApp) [75,80,82]; 3 studies used an exergaming program [17,73,85]; 3 studies used the internet, with interventions including informational websites [83,84] and tablet computers [72]; and several studies used technology to dispatch reminders to exercise and send motivational messages encouraging persistence. Specifically, studies sent text messages and made telephone calls [71,76-79,81].

The intervention duration ranged from 1 week [78] to 36 months [77]. Seven studies had interventions that lasted >3 months [59,61,71,76,77,80,82]. Only 3 studies included follow-up assessment after intervention, with durations of 6 weeks [84], 3 months [72], and 6 months [60]. Regarding intervention types, this study consisted of 12 studies supported by parents [59-61,71,72,75-77,79-81,84], 3 studies led by teachers [78,82,83], and 4 studies involving eHealth interventions directed at children [17,73,74,85].

The comparison groups included a waitlist control group (n=4) [74,79,81,84], education as usual (n=7) [17,59,75,78,80,82,85], and an additional non-eHealth intervention (n=8) [59-61,71-73,76,77]. A total of 14 studies targeted PA [17,59-61,72-75,77,78,80,81,83,85], 12 studies targeted SB [59-61,71,76-80,82,84,85], and 4 studies targeted sleep duration [71,76,81,84]. Notably, no studies examined all 3 movement behaviors.

### Meta-Analyses

#### Overview

**Figure 2:**
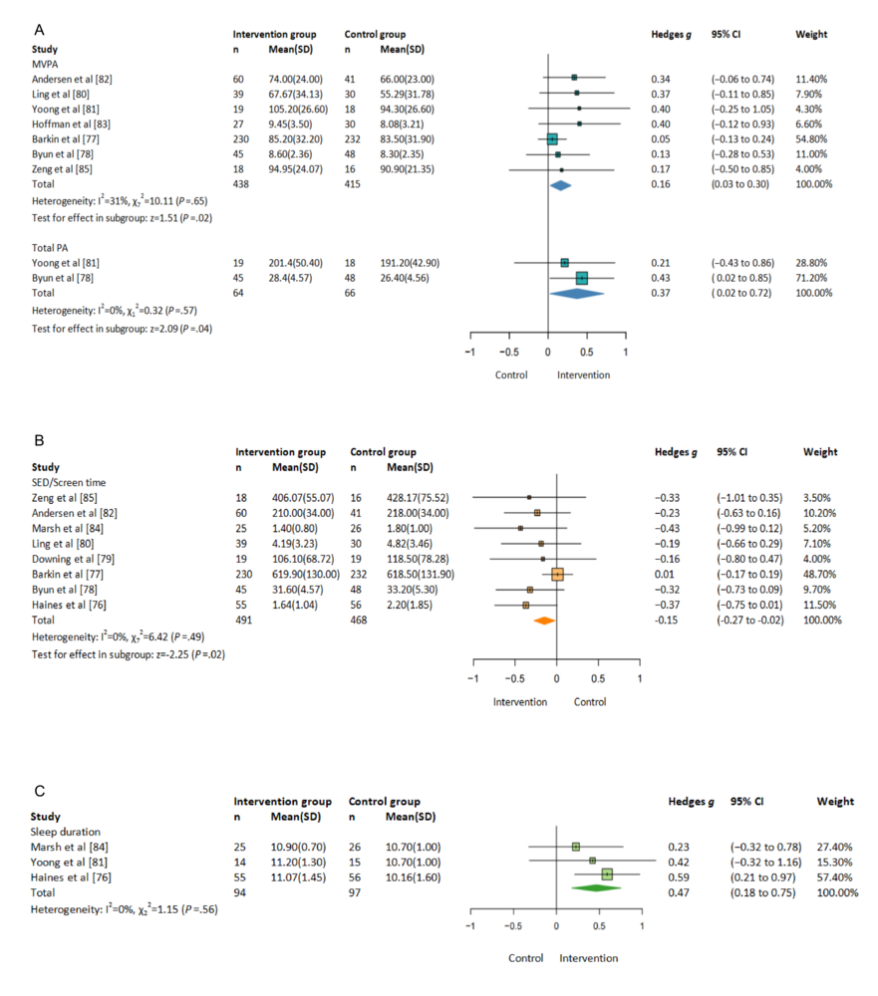
Forest plots: eHealth intervention versus control group (A) effect on moderate to vigorous physical activity (MVPA), (B) effect on sedentary time (SED), and (C) effect on sleep [76-85].

Meta-analyses demonstrated that eHealth interventions produced significant improvements in MVPA (Hedges *g*=0.16, 95% CI 0.03-0.30; *P*=.02; 7/13, 54%) and total PA (Hedges *g*=0.37, 95% CI 0.02-0.72; *P*=.04; 2/13, 15%), as shown in Figure 2A [77,78,80-83,85]. For SB outcomes, another meta-analysis showed a significant decrease (Hedges *g*=−0.15, 95% CI −0.27 to −0.02; *P*=.02; 8/13, 62%), as shown in Figure 2B [76-80,82,84,85]. Finally, meta-analysis also showed that there were significant improvements in sleep duration (Hedges *g*=0.47, 95% CI 0.18-0.75; *P*<.01; 3/13, 23%), as shown in Figure 2C [76,81,84].

Owing to the heterogeneity among the included studies, the mobile-based intervention intended to stop obesity in preschoolers (MINISTOP) project’s 3 studies solely reported the difference in pre-to-post comparison [60,61,76]. Consequently, their inclusion in the pooled analysis with other studies was deemed inappropriate. We analyzed a series of MINISTOP studies separately and presented the findings using a forest plot. The pooled analysis indicated that no significant change in MVPA (Hedges *g*=−0.03, 95% CI −0.15 to 0.09; *P*=.66; 3/6, 50%; Multimedia Appendix 10 [59-61,76-85]) was observed between the intervention and control groups. An intervention effect was found in reducing SB (Hedges *g*=0.02, 95% CI −0.13 to 0.16; *P*=.83; 3/6, 50%; Multimedia Appendix 10) immediately after the intervention, as indicated in Multimedia Appendix 10. Nonetheless, this effect was not statistically significant. All the results showed negligible heterogeneity (*I*^2^=0).

#### Subgroup Analyses and Meta-Regression

Table 2 shows the subgroup analysis and meta-regression results of MVPA and sedentary time according to study characteristics. No significant moderating effects were observed for any of the variables assessed (*P*>.05). The complete results of the subgroup analyses are presented in Multimedia Appendix 11 [59-61,76-85].

**Table 2:** Subgroup analysis and meta-regression results of MVPA^a^ and sedentary time.

		MVPA						Sedentary time				
		Studies, n	Hedges *g* (95% CI)	*I*^2^ (%)	*P* value		Studies, n		Hedges *g* (95% CI)	*I*^2^ (%)	*P* value	
Overall		7	0.16 (0.03 to 0.30)	0	.02		8		−0.15 (−0.27 to −0.02)	0	.02	
**Duration**						.59						.30
	0-3 mo	4	0.25 (−0.02 to 0.52)	25.9			4		−0.32 (−0.59 to −0.05)	22.4		
	>3 mo	3	0.17 (−0.04 to 0.38)	74.1			4		−0.13 (−0.32 to 0.05)	77.6		
**Risk of bias**						.14						.33
	Low risk	2	0.06 (−0.11 to 0.24)	58.8			4		−0.13 (−0.34 to 0.08)	67.8		
	Some concerns	5	0.3 (0.09 to 0.51)	41.2			4		−0.28 (−0.51 to −0.06)	32.2		
**Measurement**						N/A^b^						.20
	Objective	7	N/A	N/A			6		−0.1 (−0.24 to 0.04)	16.7		
	Self-reported	0	N/A	N/A			2		−0.39 (−0.70 to −0.08)	83.3		
**Type**						N/A						N/A
	Teacher focused	3	0.27 (0.02 to 0.52)	29	N/A		2		−0.28 (−0.56 to 0.01)	19.9	N/A	
	Parent supported	3	0.24 (−0.07 to 0.34)	67	.40^c^		5		−0.15 (−0.33 to 0.04)	76.6	.57^c^	
	Child centered	1	0.16 (0.03 to 0.30)	4	.80^c^		1		−0.15 (−0.27 to −0.02)	3.5	.90^c^	

^a^MVPA: moderate to vigorous physical activity.

^b^N/A: not applicable.

^c^Teacher focused studies as a reference group.

### Sensitivity Analyses and Publication Bias

Sensitivity analysis indicated that no individual study had an excessive influence on the results. The omitted meta-analytic estimates were not significantly different from those associated with the combined analysis, and all estimates were within the 95% CI. Forest plots of the sensitivity analysis for MVPA, sedentary time, and sleep are summarized in Multimedia Appendix 12 [59-61,76-85]. The significance of Egger’s test results provided evidence for asymmetry of the funnel plots (MVPA: *t_5_*=3.27; *P*=.02; Multimedia Appendix 13; sedentary time: *t_6_*=−3.37; *P*=.02; Multimedia Appendix 14). However, we could not distinguish chance from true asymmetry using the funnel plot asymmetry test because <10 studies were included in our meta-analysis [86].

### ROB of Studies

Multimedia Appendix 15 [59-61,76-85] summarizes the overall ROB assessment for all the included papers. Six studies were considered to have a low ROB [59,74,76,77,79,85], and the remaining 13 were considered to have some concerns regarding the ROB [17,60,61,71-73,75,78,80-84]. Furthermore, 7 studies did not disclose randomization methods clearly [17,72,75,78,80,82,83], so they were rated as having some concerns about random sequence generation. All studies were rated as having a low risk for the measurement of outcomes based on the use of objective measurement tools or reliable questionnaires in each study. Four studies were rated as ‘some concerns’ of reporting bias because neither published study protocols nor registered trial records were presented [72,75,78,80].

### Quality of the Evidence

The GRADE scores are shown in Multimedia Appendix 16, and we deemed the overall quality of evidence to be moderate to low. The quality of evidence for MVPA and sedentary time outcomes was rated as “moderate,” considering the low ROB, absence of heterogeneity in participants’ outcomes, and high precision in results. As eHealth interventions are often combined with other intervention approaches, all evaluations of directness were assessed as “Indirectness.” There were high imprecisions with the sample size included in the study for total PA and sleep, which were graded as “Low.”

## Discussion

### Principal Findings

This study systematically reviewed the effectiveness of eHealth interventions targeting 24-hour movement behaviors among preschool-aged children. Most studies assessed interventions aimed at increasing PA and decreasing SB. Few studies targeted sleep, and no studies have addressed a combination of all 24-hour movement behaviors. Overall, these studies showed trends supporting the effectiveness of eHealth interventions in increasing PA and sleep duration and reducing sedentary time immediately after the intervention; however, only short-term effects were found, and all trials were judged to be of low to moderate quality.

This review demonstrates a small positive effect of eHealth interventions targeting increases in preschooler’s MVPA (Hedges *g*=0.16) and total PA (Hedges *g*=0.37) immediately after the intervention. One possible explanation could be that eHealth interventions, while providing new opportunities for PA, might not be sufficient to result in significant overall activity increases. This might require expanding activity opportunities, extending new activity options, and enhancing broader activity strategies to achieve substantial benefits. Our findings echo the argument made in a previous study of young children that PA interventions had a small effect on MVPA [87]. Another meta-analysis found a positive impact of PA interventions with small to moderate effects on total PA (Hedges *g*=0.44) and moderate effects on MVPA (Hedges *g*=0.51) [29]. There is no conclusive explanation as to why MVPA and total PA were seen to have a smaller effect in our study, but this could be attributed to most interventions thus far concentrating on devising PA programs of diverse intensities without distinct objectives, including low-intensity PA, MVPA, and total PA (eg, activities such as outdoor active play and structured gross motor activity sessions in childcare environments). Moreover, our results are consistent with previous review findings that digital platforms can potentially increase PA among preschoolers [53]. Hence, future interventions should aim to optimize their effectiveness in increasing PA among young children. In addition, further research is warranted to investigate the mechanisms of the changes associated with these PA outcomes. This will help enhance the size and sustainability of the effects observed in eHealth interventions.

We found no significant improvement in MVPA for mobile app interventions (MINISTOP project). This is in contrast to a review of studies focusing on mobile apps and technologies, which highlighted the significant potential to enhance PA [88]. It is worth noting that the MINISTOP project aimed to reduce obesity as its primary outcome rather than targeting MVPA. In addition, studies concentrating solely on educating parents without implementing direct interventions for children have not achieved the desired enhancements in MVPA. Thus, we cannot draw conclusions about mobile apps because few intervention studies have used these means of communication for young children and their guardians. Given the small number of studies included in our meta-analysis, the positive, negative, and null findings of the individual studies may have attenuated the results. Thus, considering the popularity and cost-effectiveness of mobile apps in the new generation, future research should investigate the potential of using emerging and novel technologies, such as mobile health, for preschoolers.

Our meta-analysis suggests that eHealth interventions may be an effective strategy for decreasing sedentary time in preschoolers, although the magnitude of the effect was small (Hedges *g*=−0.15) and short term. Nonetheless, the significance should not be understated, given that many studies indicate that reduced sedentary time during childhood correlates with improved physical and mental health outcomes in subsequent years [16,21,89]. In the subgroup analysis, the effect of eHealth interventions on sedentary time varied depending on whether accelerometer or questionnaire measures were used. The questionnaire measures yielded higher levels of sedentary time, although this difference was not statistically significant. This observation aligns with findings from the existing literature, suggesting that questionnaire-based assessments tend to overestimate the actual sedentary time. For a more accurate evaluation of the impact of eHealth interventions, future research should consider using device-based measurement methods [90].

Interestingly, most eHealth interventions aimed to increase children’s PA and reduce sedentary time with parental support. Previous research has shown that parental and family involvement were among the key intervention components that encouraged significant improvement in children’s health behaviors and a decrease in sedentary time [91,92]. Likewise, Ha et al [49] found that parents’ physical literacy predicts children’s values toward PA, and concurrent interventions that target enhancing parents’ physical literacy for PA in the family context may be more effective in raising children’s PA values. However, our subgroup analysis showed no significant improvements in MVPA or reductions in sedentary time with the parent-supported interventions. This result also aligns with a prior review indicating that parent-directed digital interventions were ineffective in improving PA [53]. In that review, 8 studies, all published before 2020, primarily used digital platforms to convey health information and education to parents. Notably, in the wake of the COVID-19 pandemic, there has been a marked increase in research centered on leveraging technology to improve children’s PA, leading to more recent studies in 3 years [93]. Furthermore, the discourse regarding the comparative value of targeting either parents or children exclusively is not a novel debate within intervention research. In contrast to the review, our study featured a larger sample size and included a quantitative analysis of effect sizes in the interventions. These insights indicate that prevailing eHealth interventions, even with parental support, may fail to effectively engage preschoolers. Recognizing the reciprocal dynamics between parents and young children can offer insights for refining digital interventions. Therefore, preliminary research is imperative to comprehensively understand the perceptions, attitudes, and driving factors of parents. Recognizing the reciprocal dynamics between parents and young children is crucial in understanding how they influence their children’s PA and SB.

Intervention duration is also an essential component for conducting acceptable and highly effective interventions. Another subgroup analysis found that interventions with a duration of <3 months had a significantly greater effect on PA and sedentary time than those with a duration of >3 months, although the results were not significant. This notion is corroborated by another systematic review, which demonstrated the difficulty in sustaining long-term behavior change, potentially attributed to the diminishing effects of behavior change interventions mediated by digital technology [41].

The meta-analysis, involving 3 studies, revealed an immediate improvement in sleep duration following the intervention. Previous research has extensively examined the influence of sleep duration during the preschool years on physical, cognitive, and psychosocial development. For instance, the systematic review by Chaput et al [6] involving 25 studies revealed a correlation between shorter sleep duration and diminished emotion regulation in children aged 0 to 4 years. Recent findings also suggest that maintaining an extended sleep duration during the early preschool stages is significant for subsequent behavioral outcomes [24]. However, few studies have focused on effective interventions to improve sleep outcomes [45,94]. Consequently, further research is warranted to explore the impact of eHealth interventions on sleep outcomes among preschoolers.

Increasing awareness of the interconnected nature of 24-hour movement behaviors highlights their intrinsic interdependence [14]. However, none of the studies in our review specifically investigated the intervention effects on all 3 movement behaviors. Generally, conventional analytical methods do not adequately consider these indicators during analysis. Therefore, future research should explore alternative approaches, such as compositional analyses, to attain a more profound comprehension of whether an optimal equilibrium is present among SB, light PA, MVPA, and sleep [90,95,96]. Furthermore, most studies in our review examined the immediate postintervention effect. Consequently, insights into the enduring nature of alterations in 24-hour movement behaviors remain elusive. Further studies should include long-term follow-up assessments. In addition, it would be interesting to obtain more insights into the feasibility of incorporating wearable devices and apps into the design of eHealth interventions. This information could inform the design of wearables and apps that effectively enhance PA, diminish sedentary time, and enhance sleep, thereby maximizing their impact on public health. Moreover, the overall quality of the interventions was suboptimal, lacking thorough descriptions or proper execution in areas such as randomization, blinded outcome assessment, valid measurement of 24-hour movement behaviors, and adjusted differences between groups. In our meta-analysis, we observed that lower-quality studies exhibited a more pronounced positive impact on the targeted outcomes. Thus, it is essential to interpret the results cautiously, recognizing that there could be an overestimation of the effect of eHealth interventions in studies of lower quality owing to potential bias. This mirrors the findings from previous reviews on eHealth childhood PA [53] and behavior change interventions among adolescents [45].

### Strengths and Limitations

This systematic review has some strengths. First, this study is the first meta-analysis to quantitatively assess the effects of previously conducted RCTs using eHealth interventions on 24-hour movement behaviors in preschoolers. Second, the review was conducted rigorously, encompassing comprehensive terms and using an extensive systematic search strategy. We focused on robust evidence from RCT studies, assessed the quality using the GRADE approach, and adhered to a preregistered protocol. This meticulous approach reduces the heterogeneity and provides a more precise estimation of the effects.

Nonetheless, several limitations of our study should be noted. First, the quality of the studies included in this review was generally low and lacked rigorous study designs. Second, the small number of studies discerned over the decade spanned by this meta-analysis underscores the nascent state of this research domain, even considering significant technological advancements and their widespread acceptance. Third, although we systematically screened relevant electronic databases to identify studies, the search was restricted to studies published in English. Finally, the lack of evidence regarding sustained effects beyond the immediate postintervention period underscores the need for extended follow-up. Future studies should strive to elucidate strategies for maintaining the intervention effects over the preschooler’s trajectory.

### Future Research and Implications

This study highlights the significant avenues for future research. First, further research is warranted to develop eHealth interventions that yield larger effect sizes and higher quality, specifically in identifying effective 24-hour movement behaviors. It is worth noting that none of the eligible eHealth interventions addressed the comprehensive integration of 24-hour movement behaviors in preschoolers, despite the increasing recognition of the interdependence between PA, SB), and sleep. Second, many studies were conducted in Western and high-income countries, prompting the need for further exploration of the effectiveness of eHealth behavior change interventions in other country settings. Third, our study’s focus was primarily on the quantitative aspects of 24-hour movement behaviors, warranting future studies to also delve into the qualitative facets, such as motor skills and sleep quality. In addition, it is crucial to recognize the pivotal role of objective measurement tools in comprehending movement behaviors among young children. Given the sporadic and unstructured nature of preschoolers’ activities, it becomes challenging for parents and teachers to accurately discern shifts in MVPA and SB, even if they have occurred. This highlights the importance of using objective measurement tools for precise insights into these behaviors. Finally, future research in this field should prioritize broadening the focus and incorporate additional dimensions, such as physical, affective, and cognitive indicators. This approach may promote the holistic development of young children and contribute to advancements in the field of health outcomes. By considering these dimensions, researchers can also gain a comprehensive understanding of the various factors that influence children’s overall well-being and physical literacy development.

Given the multifaceted nature of intervention moderators, further research is warranted to establish optimal patterns of daily movement behaviors and to gain deeper insights into the mechanisms underlying change when addressing the amalgamation of 24-hour movement behaviors in preschoolers. Indeed, future interventions should also draw from the effective behavior change techniques used in single-behavior eHealth interventions and apply them to interventions targeting multiple healthy movement behaviors. Moreover, collaborative engagement with parents and teachers throughout both the developmental and implementation phases of these interventions will play a pivotal role in their success. In addition, capitalizing on emerging and novel technologies may offer a valuable avenue to enhance the effectiveness and feasibility of these interventions.

### Conclusions

The findings suggest that eHealth interventions may hold promise in improving 24-hour movement behaviors, particularly by increasing PA, improving sleep duration, and reducing sedentary time among preschoolers. However, these effects were relatively modest and transient and were observed primarily immediately after the intervention. Furthermore, the overall quality of the evidence was rated as moderate to low. As a result, there is a pressing need for rigorous and high-quality research endeavors to develop eHealth interventions capable of effectively enhancing both the quantity and quality of 24-hour movement behaviors simultaneously. These interventions should strive to maintain their effects over extended periods.

## Appendix

Multimedia Appendix 1 Eligibility criteria for study inclusion.

Multimedia Appendix 2 Search strategy.

Multimedia Appendix 3 Missing data processing.

Multimedia Appendix 4 Exclusion studies.

Multimedia Appendix 5 PRISMA (Preferred Reporting Items for Systematic Reviews and Meta-Analyses) checklist.

Multimedia Appendix 6 PRISMA (Preferred Reporting Items for Systematic Reviews and Meta-Analyses) abstract checklist.

Multimedia Appendix 7 Number of studies included per country and income economy.

Multimedia Appendix 8 Summary of intervention details in the included studies.

Multimedia Appendix 9 Characteristics of the included studies including physical activity, sedentary behavior, and sleep outcomes.

Multimedia Appendix 10 Forest plot of the mobile-based intervention intended to stop obesity in preschoolers (MINISTOP) results.

Multimedia Appendix 11 Forest plots of the subgroup analyses of moderate to vigorous physical activity and sedentary behavior.

Multimedia Appendix 12 Sensitive analysis.

Multimedia Appendix 13 Moderate to vigorous physical activity bias funnel.

Multimedia Appendix 14 Sedentary behavior bias funnel.

Multimedia Appendix 15 Risk of bias.

Multimedia Appendix 16 GRADE (Grading of Recommendations Assessment, Development, and Evaluation) assessment results.

## Abbreviations

GRADE: Grading of Recommendations Assessment, Development, and Evaluation
MINISTOP: mobile-based intervention intended to stop obesity in preschoolers
MVPA: moderate to vigorous physical activity
PA: physical activity
PRISMA: Preferred Reporting Items for Systematic Reviews and Meta-Analyses
RCT: randomized controlled trial
ROB: risk of bias
SB: sedentary behavior
WHO: World Health Organization

## Data Availability

The data sets generated during and analyzed during this study are available from the corresponding author on reasonable request.
