# Supplementary material for: Effects of eHealth Interventions on 24-Hour Movement Behaviors Among Preschoolers: Systematic Review and Meta-Analysis
Source: J Med Internet Res. 2024 Feb 21;26:e52905. doi: 10.2196/52905 (PMC10918543; doi:10.2196/52905)
Supplement: Multimedia Appendix 1 [file jmir_v26i1e52905_app1.docx]

**Supplementary Material 1: Eligibility criteria for study inclusion**

Inclusion Criteria:

To be eligible for inclusion in the review, studies had to meet the following criteria, according to PICOS (population, intervention, comparison, outcomes, and study design) , were as follows:

- Participants: 1)The age range of the children in the study was between 2 and 6 years or subjects mentioned in the studies were preschoolers.
- Interventions: eHealth interventions had to be delivered using. eHealth intervention, as defined as using any forms of electronic devices, delivered via at least one of the following components: wearable health and movement trackers; websites; smartphone applications; messaging services (i.e. text messaging and emails); videogames or tele-health. Single and multi-component eHealth interventions could be delivered alone or in combination with other intervention delivery methods, for example, face to face, printed materials, or exercise training sessions. The eHealth interventions could be conducted for young children with or without teachers’ or families’ support.
- Control: The controp groups included no intervention, education as usual, or an alternate evidence-based intervention not delivered via eHealth (eg, face-to-face).
- Outcome: eHealth interventions that targeted changing one or more movement behaviors (physical activity, sedentary behavior, and sleep), as defined as either primary or secondary outcomes. Valid outcomes of physical activity or sedentary behavior (i.e. no mean time (minutes) per day/week engaged in physical activity, couts of steps per day, etc.) or sedentary behavior. Sleep quality, sleep duration and other outcome characterization for sleep among young children were also included.
- Study Design: This review focussed on experimental studies with a randomization procedure. We included randomized controlled trials (RCTs), where randomization was implemented at individual or cluster level. RCTs as these are considered the gold-standard for assessing interventions; randomisation, when correctly implemented, eliminates bias in treatment assignment and limits confounding. Cross-over trials were also included if they were randomized and met the other inclusion criteria. However, only the first period of data from each arm in a cross-over trial was extracted and analyzed to avoid contamination.
- Language: The search was restricted to English-language studies.

Exclusion criteria:

Studies were excluded if they met the following criteria:

- Meta-analyses, review articles, qualitative studies, conference articles, letters, commentaries, protocols, or pilot studies; quasi-experimental studies with any randomization procedure; observational studies (e.g., cross-sectional, case control, and cohort) or expert opinions
- Control groups included participants or their guardians not using technology.
- Articles with inadequate statistical information and incomplete data were unavailable.
- Study protocol and process evaluation papers provide no intervention results, and after contact with authors, they can’t respond with some required information.

Reference:

Liberati A, Altman DG, Tetzlaff J, Mulrow C, Gøtzsche PC, Ioannidis JPA, et al. The PRISMA statement for reporting systematic reviews and meta-analyses of studies that evaluate healthcare interventions: explanation and elaboration. BMJ 2009 Jul 21;339(jul21 1):b2700
