# Supplementary material for: Effects of eHealth Interventions on 24-Hour Movement Behaviors Among Preschoolers: Systematic Review and Meta-Analysis
Source: J Med Internet Res. 2024 Feb 21;26:e52905. doi: 10.2196/52905 (PMC10918543; doi:10.2196/52905)
Supplement: Multimedia Appendix 2 [file jmir_v26i1e52905_app2.docx]

**Supplementary Material 2: Search Strategy**

**Title:** Effects of eHealth interventions on movement behaviors among preschoolers: a systematic review and meta-analysis

**Search ran:** 8 February 2023

**Databases:** Pubmed, Web of science, Cochrane Library, SPORTDiscus, OVID, Scopus

| **No.** | **Content** | **Concept** | **Searches** | **Search Fields** |
| --- | --- | --- | --- | --- |
| 1 | Population | Preschooler | preschooler OR (young AND children) OR (early AND childhood) OR (early AND years) OR kindergarten OR pre‐school OR childcare OR paediatric OR pediatric | Title, Abstract |
| 2 | Intervention | eHealth | eHealth OR e-health OR (digital health) OR (electronic health) OR mhealth OR m-health OR (mobile health) OR web OR internet OR online OR DVD-based OR smartphone OR (mobile phone) OR wearable OR (social media) OR computer OR (video gaming) OR app OR email OR telemedicine OR e-learning OR elearning OR texting OR SMS OR (text messaging) OR Facebook OR wechat OR WhatsApp OR YouTube OR skype OR Instagram OR twitter OR Tumblr OR Snapchat OR Reddit OR Myspace OR Xiaohongshu OR weibo OR Tiktok | Title, Abstract |
| 3 | Outcomes | Physical activity | Sport* OR (physical activit*) OR exercise* OR aerobic* OR outdoor* OR playground* OR active recreation* OR walk* OR bicycl* OR biking OR crawl* OR swim* OR soccer OR Dancing OR jumping OR jogging OR running OR game* | Title, Abstract |
| 4 |  | Sedentary behavior | (sedentary behavior*) OR (sedentary behaviour*) OR (sedentary lifestyle*) OR (screen time*) OR (television viewing) OR (video game*) OR (screen-based media) OR sitting OR (Stationary behavior) OR (sitting time) OR (physical inactivit*) | Title, Abstract |
| 5 |  | Sleep | Sleep* OR Bedtime* OR (sleep timing) OR Polysomnography OR Insomnia OR (Time in bed) OR Wake* OR Awake* OR Waking OR REM OR (rapid eye movement) OR (sleep quality) OR (sleep latency) OR (sleep efficiency) OR (sleep duration) OR (sleep hygiene) OR (sleep satisfaction) OR (sleep routine) OR (sleep onset) OR (sleep diary) OR (sleep practice) OR (sleep habit) | Title, Abstract |
| 6 | Outcome | Movement behaviour | #3 OR #4 OR #5 |  |
| 7 | Study Design | RCT | RCT OR (randomized clinical trial) OR (randomized trial) OR (randomized controlled trial) OR randomization OR randomly OR randomized | All fields |
| 8 | Searching |  | #1 AND #2 AND #6 AND #7 |  |

**1. Database search in Pubmed (title/abstract)**

**Results:** 2042

**Searching algorithm:**

("preschooler"[Title/Abstract] OR "young children"[Title/Abstract] OR "early childhood"[Title/Abstract] OR "early years"[Title/Abstract] OR "kindergarten"[Title/Abstract] OR "pre-school"[Title/Abstract] OR "childcare"[Title/Abstract] OR "paediatric"[Title/Abstract] OR "pediatric"[Title/Abstract]) AND ("eHealth"[Title/Abstract] OR "e-health"[Title/Abstract] OR "digital health"[Title/Abstract] OR "electronic health"[Title/Abstract] OR "mhealth"[Title/Abstract] OR "m-health"[Title/Abstract] OR "mobile health"[Title/Abstract] OR "web"[Title/Abstract] OR "internet"[Title/Abstract] OR "online"[Title/Abstract] OR "DVD-based"[Title/Abstract] OR "smartphone"[Title/Abstract] OR "mobile phone"[Title/Abstract] OR "wearable"[Title/Abstract] OR "social media"[Title/Abstract] OR "computer"[Title/Abstract] OR "video gaming"[Title/Abstract] OR "app"[Title/Abstract] OR "email"[Title/Abstract] OR "telemedicine"[Title/Abstract] OR "e-learning"[Title/Abstract] OR "elearning"[Title/Abstract] OR "texting"[Title/Abstract] OR "SMS"[Title/Abstract] OR "text messaging"[Title/Abstract] OR "Facebook"[Title/Abstract] OR "wechat"[Title/Abstract] OR "WhatsApp"[Title/Abstract] OR "YouTube"[Title/Abstract] OR "skype"[Title/Abstract] OR "Instagram"[Title/Abstract] OR "twitter"[Title/Abstract] OR "Tumblr"[Title/Abstract] OR "Snapchat"[Title/Abstract] OR "Reddit"[Title/Abstract] OR "Myspace"[Title/Abstract] OR "Xiaohongshu"[Title/Abstract] OR "weibo"[Title/Abstract] OR "Tiktok"[Title/Abstract]) AND ("sport*"[Title/Abstract] OR "physical activit*"[Title/Abstract] OR "exercise*"[Title/Abstract] OR "aerobic*"[Title/Abstract] OR "outdoor*"[Title/Abstract] OR "playground*"[Title/Abstract] OR "active recreation*"[Title/Abstract] OR "walk*"[Title/Abstract] OR "bicycl*"[Title/Abstract] OR "biking"[Title/Abstract] OR "crawl*"[Title/Abstract] OR "swim*"[Title/Abstract] OR "soccer"[Title/Abstract] OR "Dancing"[Title/Abstract] OR "jumping"[Title/Abstract] OR "jogging"[Title/Abstract] OR "running"[Title/Abstract] OR "game*"[Title/Abstract] OR "sedentary behavior*"[Title/Abstract] OR "sedentary behaviour*"[Title/Abstract] OR "sedentary lifestyle*"[Title/Abstract] OR "screen time*"[Title/Abstract] OR "television viewing"[Title/Abstract] OR "video game*"[Title/Abstract] OR "screen based media"[Title/Abstract] OR "sitting"[Title/Abstract] OR "stationary behavior"[Title/Abstract] OR "sitting time"[Title/Abstract] OR "physical inactivit*"[Title/Abstract] OR "sleep*"[Title/Abstract] OR "bedtime*"[Title/Abstract] OR "sleep timing"[Title/Abstract] OR "Polysomnography"[Title/Abstract] OR "Insomnia"[Title/Abstract] OR "time in bed"[Title/Abstract] OR "wake*"[Title/Abstract] OR "awake*"[Title/Abstract] OR "Waking"[Title/Abstract] OR "REM"[Title/Abstract] OR "rapid eye movement"[Title/Abstract] OR "sleep quality"[Title/Abstract] OR "sleep latency"[Title/Abstract] OR "sleep efficiency"[Title/Abstract] OR "sleep duration"[Title/Abstract] OR "sleep hygiene"[Title/Abstract] OR "sleep satisfaction"[Title/Abstract] OR "sleep routine"[Title/Abstract] OR "sleep onset"[Title/Abstract] OR "sleep diary"[Title/Abstract] OR "sleep practice"[Title/Abstract] OR "sleep habit"[Title/Abstract] OR "OR"[Title/Abstract]) AND ("RCT"[All Fields] OR ("randomized controlled trial"[Publication Type] OR "randomized controlled trials as topic"[MeSH Terms] OR "randomized clinical trial"[All Fields] OR "randomised clinical trial"[All Fields]) OR (("random allocation"[MeSH Terms] OR ("random"[All Fields] AND "allocation"[All Fields]) OR "random allocation"[All Fields] OR "randomization"[All Fields] OR "randomized"[All Fields] OR "random"[All Fields] OR "randomisation"[All Fields] OR "randomisations"[All Fields] OR "randomise"[All Fields] OR "randomised"[All Fields] OR "randomising"[All Fields] OR "randomizations"[All Fields] OR "randomize"[All Fields] OR "randomizes"[All Fields] OR "randomizing"[All Fields] OR "randomness"[All Fields] OR "randoms"[All Fields]) AND ("clinical trials as topic"[MeSH Terms] OR ("clinical"[All Fields] AND "trials"[All Fields] AND "topic"[All Fields]) OR "clinical trials as topic"[All Fields] OR "trial"[All Fields] OR "trial s"[All Fields] OR "trialed"[All Fields] OR "trialing"[All Fields] OR "trials"[All Fields])) OR ("randomized controlled trial"[Publication Type] OR "randomized controlled trials as topic"[MeSH Terms] OR "randomized controlled trial"[All Fields] OR "randomised controlled trial"[All Fields]) OR ("random allocation"[MeSH Terms] OR ("random"[All Fields] AND "allocation"[All Fields]) OR "random allocation"[All Fields] OR "randomization"[All Fields] OR "randomized"[All Fields] OR "random"[All Fields] OR "randomisation"[All Fields] OR "randomisations"[All Fields] OR "randomise"[All Fields] OR "randomised"[All Fields] OR "randomising"[All Fields] OR "randomizations"[All Fields] OR "randomize"[All Fields] OR "randomizes"[All Fields] OR "randomizing"[All Fields] OR "randomness"[All Fields] OR "randoms"[All Fields]) OR "randomly"[All Fields] OR ("random allocation"[MeSH Terms] OR ("random"[All Fields] AND "allocation"[All Fields]) OR "random allocation"[All Fields] OR "randomization"[All Fields] OR "randomized"[All Fields] OR "random"[All Fields] OR "randomisation"[All Fields] OR "randomisations"[All Fields] OR "randomise"[All Fields] OR "randomised"[All Fields] OR "randomising"[All Fields] OR "randomizations"[All Fields] OR "randomize"[All Fields] OR "randomizes"[All Fields] OR "randomizing"[All Fields] OR "randomness"[All Fields] OR "randoms"[All Fields]))

**2. Database search in Web of science (title/abstract)**

**Results:** 1196

**Searching algorithm:**

<https://www.webofscience.com/wos/woscc/summary/2b0de6b1-2edb-48ee-9a25-2c150f223657-6e80a9bb/relevance/1>

**Title (31)**

TI=(preschooler OR young children OR early childhood OR early years OR kindergarten OR pre‐school OR childcare OR paediatric OR pediatric) AND TI=(eHealth OR e-health OR digital health OR electronic health OR mhealth OR m-health OR mobile health OR web OR internet OR online OR DVD-based OR smartphone OR mobile phone OR wearable OR social media OR computer OR video gaming OR app OR email OR telemedicine OR e-learning OR elearning OR texting OR SMS OR text messaging OR Facebook OR wechat OR WhatsApp OR YouTube OR skype OR Instagram OR twitter OR Tumblr OR Snapchat OR Reddit OR Myspace OR Xiaohongshu OR weibo or Tiktok) AND TI=(Sport* OR physical activit* OR exercise* OR aerobic* OR outdoor* OR playground* OR active recreation* OR walk* OR bicycl* OR biking OR crawl* OR swim* OR soccer OR Dancing OR jumping OR jogging OR running OR game* OR sedentary behavior* OR sedentary behaviour* OR sedentary lifestyle* OR screen time* OR television viewing OR video game* OR screen-based media OR sitting OR Stationary behavior OR sitting time OR physical inactivit* OR Sleep* OR Bedtime* OR sleep timing OR Polysomnography OR Insomnia OR Time in bed OR Wake* OR Awake* OR Waking OR REM OR rapid eye movement OR sleep quality OR sleep latency OR sleep efficiency OR sleep duration OR sleep hygiene OR sleep satisfaction OR sleep routine OR sleep onset OR sleep diary OR sleep practice OR sleep habit) AND ALL=(RCT OR randomized clinical trial OR randomized trial OR randomized controlled trial OR randomization OR randomly OR randomized )

**Abstract (1186)**

AB=(preschooler OR young children OR early childhood OR early years OR kindergarten OR pre‐school OR childcare OR paediatric OR pediatric) AND AB=(eHealth OR e-health OR digital health OR electronic health OR mhealth OR m-health OR mobile health OR web OR internet OR online OR DVD-based OR smartphone OR mobile phone OR wearable OR social media OR computer OR video gaming OR app OR email OR telemedicine OR e-learning OR elearning OR texting OR SMS OR text messaging OR Facebook OR wechat OR WhatsApp OR YouTube OR skype OR Instagram OR twitter OR Tumblr OR Snapchat OR Reddit OR Myspace OR Xiaohongshu OR weibo or Tiktok) AND AB=(Sport* OR physical activit* OR exercise* OR aerobic* OR outdoor* OR playground* OR active recreation* OR walk* OR bicycl* OR biking OR crawl* OR swim* OR soccer OR Dancing OR jumping OR jogging OR running OR game* OR sedentary behavior* OR sedentary behaviour* OR sedentary lifestyle* OR screen time* OR television viewing OR video game* OR screen-based media OR sitting OR Stationary behavior OR sitting time OR physical inactivit* OR Sleep* OR Bedtime* OR sleep timing OR Polysomnography OR Insomnia OR Time in bed OR Wake* OR Awake* OR Waking OR REM OR rapid eye movement OR sleep quality OR sleep latency OR sleep efficiency OR sleep duration OR sleep hygiene OR sleep satisfaction OR sleep routine OR sleep onset OR sleep diary OR sleep practice OR sleep habit) AND ALL=(RCT OR randomized clinical trial OR randomized trial OR randomized controlled trial OR randomization OR randomly OR randomized )

**3. Database search in Cochrane Library (Title, abstract, keywords)**

**Results:** 226

**Searching algorithm:**

(RCT OR randomized clinical trial OR randomized trial OR randomized controlled trial OR randomization OR randomly OR randomized in All Text) AND (preschooler OR young children OR early childhood OR early years OR kindergarten OR pre‐school OR childcare OR paediatric OR pediatric in Title Abstract Keyword) AND eHealth OR e-health OR digital health OR electronic health OR mhealth OR m-health OR mobile health OR web OR internet OR online OR DVD-based OR smartphone OR mobile phone OR wearable OR social media OR computer OR video gaming OR app OR email OR telemedicine OR e-learning OR elearning OR texting OR SMS OR text messaging OR Facebook OR wechat OR WhatsApp OR YouTube OR skype OR Instagram OR twitter OR Tumblr OR Snapchat OR Reddit OR Myspace OR Xiaohongshu OR weibo or Tiktok in Title Abstract Keyword AND Sport* OR physical activit* OR exercise* OR aerobic* OR outdoor* OR playground* OR active recreation* OR walk* OR bicycl* OR biking OR crawl* OR swim* OR soccer OR Dancing OR jumping OR jogging OR running OR game* OR sedentary behavior* OR sedentary behaviour* OR sedentary lifestyle* OR screen time* OR television viewing OR video game* OR screen-based media OR sitting OR Stationary behavior OR sitting time OR physical inactivit* OR Sleep* OR Bedtime* OR sleep timing OR Polysomnography OR Insomnia OR Time in bed OR Wake* OR Awake* OR Waking OR REM OR rapid eye movement OR sleep quality OR sleep latency OR sleep efficiency OR sleep duration OR sleep hygiene OR sleep satisfaction OR sleep routine OR sleep onset OR sleep diary OR sleep practice OR sleep habit in Title Abstract Keyword

**4. Database search in SPORTDiscus**

**Results:** 53

**Searching algorithm:**

#S1: 18,170

TI ( preschooler OR young children OR early childhood OR early years OR kindergarten OR pre‐school OR childcare OR paediatric OR pediatric ) OR AB ( preschooler OR young children OR early childhood OR early years OR kindergarten OR pre‐school OR childcare OR paediatric OR pediatric )

#S2: 64,841

TI ( eHealth OR e-health OR digital health OR electronic health OR mhealth OR m-health OR mobile health OR web OR internet OR online OR DVD-based OR smartphone OR mobile phone OR wearable OR social media OR computer OR video gaming OR app OR email OR telemedicine OR e-learning OR elearning OR texting OR SMS OR text messaging OR Facebook OR wechat OR WhatsApp OR YouTube OR skype OR Instagram OR twitter OR Tumblr OR Snapchat OR Reddit OR Myspace OR Xiaohongshu OR weibo or Tiktok ) OR AB ( eHealth OR e-health OR digital health OR electronic health OR mhealth OR m-health OR mobile health OR web OR internet OR online OR DVD-based OR smartphone OR mobile phone OR wearable OR social media OR computer OR video gaming OR app OR email OR telemedicine OR e-learning OR elearning OR texting OR SMS OR text messaging OR Facebook OR wechat OR WhatsApp OR YouTube OR skype OR Instagram OR twitter OR Tumblr OR Snapchat OR Reddit OR Myspace OR Xiaohongshu OR weibo or Tiktok )

#S3: 790,317

TI ( Sport* OR physical activit* OR exercise* OR aerobic* OR outdoor* OR playground* OR active recreation* OR walk* OR bicycl* OR biking OR crawl* OR swim* OR soccer OR Dancing OR jumping OR jogging OR running OR game* OR sedentary behavior* OR sedentary behaviour* OR sedentary lifestyle* OR screen time* OR television viewing OR video game* OR screen-based media OR sitting OR Stationary behavior OR sitting time OR physical inactivit* OR Sleep* OR Bedtime* OR sleep timing OR Polysomnography OR Insomnia OR Time in bed OR Wake* OR Awake* OR Waking OR REM OR rapid eye movement OR sleep quality OR sleep latency OR sleep efficiency OR sleep duration OR sleep hygiene OR sleep satisfaction OR sleep routine OR sleep onset OR sleep diary OR sleep practice OR sleep habit ) OR AB ( Sport* OR physical activit* OR exercise* OR aerobic* OR outdoor* OR playground* OR active recreation* OR walk* OR bicycl* OR biking OR crawl* OR swim* OR soccer OR Dancing OR jumping OR jogging OR running OR game* OR sedentary behavior* OR sedentary behaviour* OR sedentary lifestyle* OR screen time* OR television viewing OR video game* OR screen-based media OR sitting OR Stationary behavior OR sitting time OR physical inactivit* OR Sleep* OR Bedtime* OR sleep timing OR Polysomnography OR Insomnia OR Time in bed OR Wake* OR Awake* OR Waking OR REM OR rapid eye movement OR sleep quality OR sleep latency OR sleep efficiency OR sleep duration OR sleep hygiene OR sleep satisfaction OR sleep routine OR sleep onset OR sleep diary OR sleep practice OR sleep habit )

#S4: 144,642

TX RCT OR randomized clinical trial OR randomized trial OR randomized controlled trial OR randomization OR randomly OR randomized

#S1 AND #S2 AND #S3 AND #S4 = 53

**5. Database search in OVID**

**Results:** 490

**Searching algorithm:**

<https://easyaccess.lib.cuhk.edu.hk/login?url=http://ovidsp.ovid.com/ovidweb.cgi?T=JS&NEWS=N&PAGE=main&SHAREDSEARCHID=4c4JVxcdqbNUpiFX3Z3jA7WtyU6EzZ9SKMv0RDU2u421027aaPM1yjRTpf7R35iMl>

(preschooler or young children or early childhood or early years or kindergarten or preschool or childcare or paediatric or pediatric).ab,ti. AND (eHealth or ehealth or digital health or electronic health or mhealth or mobile health or web or internet or online or DVD or smartphone or mobile phone or wearable or social media or computer or video gaming or app or email or telemedicine or elearning or texting or SMS or text messaging or Facebook or wechat or WhatsApp or YouTube or skype or Instagram or twitter or Tumblr or Snapchat or Reddit or Myspace or Xiaohongshu or weibo or Tiktok).ab,ti. AND (Sport* or physical activit* or exercise* or aerobic* or outdoor* or playground* or active recreation* or walk* or bicycl* or biking or crawl* or swim* or soccer or Dancing or jumping or jogging or running or game* or sedentary behavior* or sedentary behaviour* or sedentary lifestyle* or screen time* or television viewing or video game* or screen-based media or sitting or Stationary behavior or sitting time or physical inactivit* or Sleep* or Bedtime* or sleep timing or Polysomnography or Insomnia or Time in bed or Wake* or Awake* or Waking or REM or rapid eye movement or sleep quality or sleep latency or sleep efficiency or sleep duration or sleep hygiene or sleep satisfaction or sleep routine or sleep onset or sleep diary or sleep practice or sleep habit).ab,ti. AND (RCT or randomized clinical trial or randomized trial or randomized controlled trial or randomization or randomly or randomized).af.

**6. Database search in Scopus**

**Results:** 3133

**Searching algorithm:**

( TITLE-ABS-KEY ( preschooler OR ( young AND children ) OR ( early AND childhood ) OR ( early AND years ) OR kindergarten OR pre‐school OR childcare OR paediatric OR pediatric ) ) AND ( TITLE-ABS-KEY ( ehealth OR e-health OR ( digital AND health ) OR ( electronic AND health ) OR mhealth OR m-health OR ( mobile AND health ) OR web OR internet OR online OR dvd-based OR smartphone OR ( mobile AND phone ) OR wearable OR ( social AND media ) OR computer OR ( video AND gaming ) OR app OR email OR telemedicine OR e-learning OR elearning OR texting OR sms OR ( text AND messaging ) OR facebook OR wechat OR whatsapp OR youtube OR skype OR instagram OR twitter OR tumblr OR snapchat OR reddit OR myspace OR xiaohongshu OR weibo OR tiktok ) ) AND ( ( TITLE-ABS-KEY ( sport* OR ( physical AND activit* ) OR exercise* OR aerobic* OR outdoor* OR playground* OR active AND recreation* OR walk* OR bicycl* OR biking OR crawl* OR swim* OR soccer OR dancing OR jumping OR jogging OR running OR game* ) ) OR ( TITLE-ABS-KEY ( ( sedentary AND behavior* ) OR ( sedentary AND behaviour* ) OR ( sedentary AND lifestyle* ) OR ( screen AND time* ) OR ( television AND viewing ) OR ( video AND game* ) OR ( screen-based AND media ) OR sitting OR ( stationary AND behavior ) OR ( sitting AND time ) OR ( physical AND inactivit* ) ) ) OR ( TITLE-ABS-KEY ( sleep* OR bedtime* OR ( sleep AND timing ) OR polysomnography OR insomnia OR ( time AND in AND bed ) OR wake* OR awake* OR waking OR rem OR ( rapid AND eye AND movement ) OR ( sleep AND quality ) OR ( sleep AND latency ) OR ( sleep AND efficiency ) OR ( sleep AND duration ) OR ( sleep AND hygiene ) OR ( sleep AND satisfaction ) OR ( sleep AND routine ) OR ( sleep AND onset ) OR ( sleep AND diary ) OR ( sleep AND practice ) OR ( sleep AND habit ) ) ) ) AND ( ALL ( rct OR ( randomized AND clinical AND trial ) OR ( randomized AND trial ) OR ( randomized AND controlled AND trial ) OR randomization OR randomly OR randomized ) )

All= 7140

Duplicates: 1492+269+=1761

Remove duplicates: 5879
