# Supplementary material for: Effects of eHealth Interventions on 24-Hour Movement Behaviors Among Preschoolers: Systematic Review and Meta-Analysis
Source: J Med Internet Res. 2024 Feb 21;26:e52905. doi: 10.2196/52905 (PMC10918543; doi:10.2196/52905)
Supplement: Multimedia Appendix 3 [file jmir_v26i1e52905_app3.docx]

**Missing data processing**

Our outcomes were physical activity (accelerometer and self-report, min per day), screen time (h per day spent using screens—eg, television, tablets, computers, or smartphones), sedentary behaviour (h per day spent sitting or lying down), and sleep duration (h/day). Considering the different outcomes in the studies, standardized mean differences (SMD) of pre-post intervention were calculated and given weight by its inverse variance.

Missing data were handled as described in the corresponding chapters of the Cochrane Handbook. If 95% CI was reported instead of SD or SE, then the SD was calculated as described using the following formula:

$$\mathrm{SD}=\sqrt{N}\times(upper limit-lower limit) /3.92$$

If SE was reported instead of SD, then this was converted to SD using the formula: $SD=SE\times\sqrt{N}$.

But if some studies were with the change of baseline and post-intervention data, or there are significant differences in their baseline data. ^1-3^ We used the within-group difference in means and their SDs for intervention and control groups to calculate the effect size. But if no SDs for the change between baseline and follow-up were available, they were calculated using the following formula:

$$\mathrm{SD}_{E,change}=\sqrt{\mathrm{SD}_{E,change}^{2}+\mathrm{SD}_{E,final}^{2}-(2\times Corr\times\mathrm{SD}_{E,baseline}\times\mathrm{SD}_{E,final})}$$

We excluded studies from the meta-analysis that did not provide SD, SE, or 95% CI (and whose authors did not respond to our request for these data).

1. Alexandrou C, Henriksson H, Henström M, et al. Effectiveness of a Smartphone App (MINISTOP 2.0) integrated in primary child health care to promote healthy diet and physical activity behaviors and prevent obesity in preschool-aged children: randomized controlled trial. *International Journal of Behavioral Nutrition and Physical Activity*. 2023/02/21 2023;20(1):22. doi:10.1186/s12966-023-01405-5

2. Delisle Nystrom C, Sandin S, Henriksson P, Henriksson H, Maddison R, Lof M. A 12-month follow-up of a mobile-based (mHealth) obesity prevention intervention in pre-school children: the MINISTOP randomized controlled trial. *BMC public health*. 2018;18(1):658. doi:<https://dx.doi.org/10.1186/s12889-018-5569-4>

3. Nyström CD, Sandin S, Henriksson P, et al. Mobile-based intervention intended to stop obesity in preschool-aged children: The MINISTOP randomized controlled trial. Article. *American Journal of Clinical Nutrition*. 2017;105(6):1327-1335. doi:10.3945/ajcn.116.150995
