# Supplementary material for: Effects of eHealth Interventions on 24-Hour Movement Behaviors Among Preschoolers: Systematic Review and Meta-Analysis
Source: J Med Internet Res. 2024 Feb 21;26:e52905. doi: 10.2196/52905 (PMC10918543; doi:10.2196/52905)
Supplement: Multimedia Appendix 4 [file jmir_v26i1e52905_app4.docx]

**Supplementary Material 4: List of excluded studies with reasons for exclusion (n=46)**

| **Study** | **Reference** | **Excluded Reason** | **Specific information** |
| --- | --- | --- | --- |
| Benjamin 2008 | Benjamin SE, Tate DF, Bangdiwala SI, Neelon BH, Ammerman AS, Dodds JM, Ward DS: Preparing child care health consultants to address childhood overweight: A randomized controlled trial comparing web to in-person training. *Maternal and Child Health Journal 2008, 12*(5):662-669. | Ineligible participants | not preschool children |
| Delisle 2015 | Delisle, C., Sandin, S., Forsum, E., Henriksson, H., Trolle-Lagerros, Y., Larsson, C., Maddison, R., Ortega, F. B., Ruiz, J. R., Silfvernagel, K., Timpka, T., & Lof, M. (2015). A web- and mobile phone-based intervention to prevent obesity in 4-year-olds (MINISTOP): a population-based randomized controlled trial. BMC Public Health, 15, 95. https://doi.org/https://dx.doi.org/10.1186/s12889-015-1444-8 | Study protocol |  |
| Ek 2019 | Ek A, Delisle Nyström C, Chirita-Emandi A, Tur JA, Nordin K, Bouzas C, Argelich E, Martínez JA, Frost G, Garcia-Perez I et al: A randomized controlled trial for overweight and obesity in preschoolers: the More and Less Europe study - an intervention within the STOP project. *BMC Public Health 2019, 19*(1):945. | Study protocol |  |
| Gago 2023 | Gago C, Aftosmes-Tobio A, Beckerman-Hsu JP, Oddleifson C, Garcia EA, Lansburg K, Figueroa R, Yu X, Kitos N, Torrico M et al: Evaluation of a cluster-randomized controlled trial: Communities for Healthy Living, family-centered obesity prevention program for Head Start parents and children. *International Journal of Behavioral Nutrition and Physical Activity 2023, 20*(1). | Ineligible intervention | not eHealth intervention |
| Ghofranipour 2022 | Ghofranipour, F., Hamzavi Zarghani, N., Mohammadi, E., Mehrizi, A. A. H., Tavousi, M., De Craemer, M., & Cardon, G. (2022). An internet-based educational intervention for mothers targeting preschoolers’ weight management promotion (PWMP): a pilot study. *BMC Public Health, 22*(1), Article 2220. | Ineligible study design | pre-post (not RCT) |
| Haines 2018 | Haines J, Douglas S, Mirotta JA, O’Kane C, Breau R, Walton K, Krystia O, Chamoun E, Annis A, Darlington GA et al: Guelph Family Health Study: pilot study of a home-based obesity prevention intervention. *Canadian Journal of Public Health 2018, 109*(4):549-560. | Ineligible comparator | Both the control and intervention groups use eHealth, and family visits are the intervention. |
| Hammersley 2019 | Hammersley, M. L., Okely, A. D., Batterham, M. J., & Jones, R. A. (2019). Investigating the mediators and moderators of child body mass index change in the Time2bHealthy childhood obesity prevention program for parents of preschool-aged children. *Public Health, 173*, 50-57. | Ineligible comparator | Both the control and intervention groups use eHealth. |
| Hammersley 2019 | Hammersley, M. L., Okely, A. D., Batterham, M. J., & Jones, R. A. (2019). An internet-based childhood obesity prevention program (TIMe2bhealthy) for parents of preschool-aged children: Randomized controlled trial. *Journal of Medical Internet Research, 21*(2). | Ineligible comparator | Both the control and intervention groups use eHealth. |
| Hammersley 2020 | Hammersley, M. L., Okely, A. D., Batterham, M. J., & Jones, R. A. (2020). Can Parental Engagement in Social Media Enhance Outcomes of an Online Healthy Lifestyle Program for Preschool-Aged Children? *Health Communication, 35*(9), 1162-1171. | Ineligible comparator | Both the control and intervention groups use eHealth. |
| Hammersley 2020 | Hammersley ML, Wyse RJ, Jones RA, Wolfenden L, Yoong S, Stacey F, Eckermann S, Okely AD, Innes-Hughes C, Li V et al: Translation of two healthy eating and active living support programs for parents of 2-6 year old children: A parallel partially randomised preference trial protocol (the 'time for healthy habits' trial). *BMC Public Health 2020, 20*(1). | Study protocol |  |
| Hauser 2010 | Hauser SI, Goldberg JP, Wilde P, Bers M, Ioannone L, Economos CD: Comparison of Online and Face-to-Face Dissemination of a Theory-Based After School Nutrition and Physical Activity Training and Curriculum. *Journal of Health Communication 2010, 15*(8):859-879. | Ineligible outcomes | no targeted movement behaviors |
| Heerman 2018 | Heerman WJ, Burgess LE, Escarfuller J, Teeters L, Slesur L, Liu J, Qi A, Samuels LR, Singer-Gabella M: Competency Based Approach to Community Health (COACH): The methods of a family-centered, community-based, individually adaptive obesity randomized trial for pre-school child-parent pairs. *Contemporary Clinical Trials 2018*, 73:1-7. | Study protocol |  |
| Johansson 2020 | Johansson L, Hagman E, Danielsson P: A novel interactive mobile health support system for pediatric obesity treatment: a randomized controlled feasibility trial. *BMC Pediatr 2020, 20*(1):447. | Ineligible participants | Participants are 5–12 years |
| Jouret 2009 | Jouret B, Ahluwalia N, Dupuy M, Cristini C, Negre-Pages L, Grandjean H, Tauber M: Prevention of overweight in preschool children: results of kindergarten-based interventions. *International Journal of Obesity 2009, 33*(10):1075-1083. | Ineligible outcomes | no targeted movement behaviors |
| Kitsaras 2022 | Kitsaras G, Pretty IA, Allan J: Bedtime Routines Intervention for Children (BRIC) project: results from a non-randomised feasibility, proof-of concept study. *Pilot and Feasibility Studies 2022, 8*(1). | Ineligible study design | pre-post (not RCT) |
| Knowlden 2018 | Knowlden, A. P., & Conrad, E. (2018). Two-Year Outcomes of the Enabling Mothers to Prevent Pediatric Obesity Through Web-Based Education and Reciprocal Determinism (EMPOWER) Randomized Control Trial. *Health Education and Behavior, 45*(2), 262-276. | Ineligible comparator | Both the control and intervention groups use eHealth. |
| Latomme 2017 | Latomme J, Cardon G, De Bourdeaudhuij I, Iotova V, Koletzko B, Socha P, Moreno L, Androutsos O, Manios Y, De Craemer M: Effect and process evaluation of a kindergarten-based, family-involved intervention with a randomized cluster design on sedentary behaviour in 4- to 6- year old European preschool children: The ToyBox-study. *PLoS One 2017, 12*(4):e0172730. | Ineligible intervention | not eHealth intervention |
| Ling 2019 | Ling, J., Zahry, N. R., & Robbins, L. B. (2019). Dose-Response Relationship in a Healthy Habits Study for Head Start Preschoolers. *Nursing research, 68*(4), 329-335. | Duplicate | A paper describing the same project and participants has been included |
| Malow 2021 | Malow, B. A., Galion, A., Lu, F., Kennedy, N., Lawrence, C. E., Tassone, A., O'Neal, L., Wilson, T. M., Parker, R. A., Harris, P. A., & Neumeyer, A. M. (2021). A REDCap-based Model for Online Interventional Research: Parent Sleep Education in Autism. *Journal of Clinical and Translational Science.* | Ineligible participants | participants aged 2–10, 11 months |
| Michels 2015 | Michels, N., de Henauw, S., Eiben, G., Hadjigeorgiou, C., Hense, S., Hunsberger, M., Konstabel, K., Molnár, D., Moreno, L. A., Siani, A., de Bourdeaudhuij, I., & Pigeot, I. (2015). Effect of the IDEFICS multilevel obesity prevention on children's sleep duration. *Obesity Reviews, 16*, 68-77. | Ineligible participants | children were 2–9.9 years of age |
| Miguel-Berges 2017 | Miguel-Berges ML, Zachari K, Santaliestra-Pasias AM, Mouratidou T, Androutsos O, Iotova V, Galcheva S, De Craemer M, Cardon G, Koletzko B et al: Clustering of energy balance-related behaviours and parental education in European preschool children: The ToyBox study. *British Journal of Nutrition 2017, 118*(12):1089-1096. | Ineligible intervention | not eHealth intervention |
| Militello 2016 | Militello L, Melnyk BM, Hekler EB, Small L, Jacobson D: Automated Behavioral Text Messaging and Face-to-Face Intervention for Parents of Overweight or Obese Preschool Children: Results From a Pilot Study. *Jmir Mhealth and Uhealth 2016, 4*(1):209-222. | Ineligible study design | pre-post (not RCT) |
| Nyberg 2015 | Nyberg, G., Sundblom, E., Norman, Å., Bohman, B., Hagberg, J., & Elinder, L. S. (2015). Effectiveness of a universal parental support programme to promote healthy dietary habits and physical activity and to prevent overweight and obesity in 6-year-old children: The healthy school start study, a cluster-randomised controlled trial. *PLoS One, 10*(2). | Ineligible intervention | not eHealth intervention |
| Penalvo 2013 | Penalvo JL, Santos-Beneit G, Sotos-Prieto M, Martinez R, Rodriguez C, Franco M, Lopez-Romero P, Pocock S, Redondo J, Fuster V: A cluster randomized trial to evaluate the efficacy of a school-based behavioral intervention for health promotion among children aged 3 to 5. *BMC public health 2013, 13*:656. | Study protocol |  |
| Po'e 2013 | Po'e EK, Heerman WJ, Mistry RS, Barkin SL: Growing Right Onto Wellness (GROW): A family-centered, community-based obesity prevention randomized controlled trial for preschool child-parent pairs. *Contemporary Clinical Trials 2013, 36*(2):436-449. | Study protocol |  |
| Raat 2013 | Raat H, Struijk MK, Remmers T, Vlasblom E, van Grieken A, Broeren SM, te Velde SJ, Beltman M, Boere-Boonekamp MM, L'Hoir MP: Primary prevention of overweight in preschool children, the BeeBOFT study (breastfeeding, breakfast daily, outside playing, few sweet drinks, less TV viewing): design of a cluster randomized controlled trial. *BMC public health 2013, 13*:974. | Study protocol |  |
| Sadeghi 2022 | Sadeghi S, Pouretemad HR, Shalani B: Internet-based versus face-to-face intervention training for parents of young children with excessive screen-time and autism spectrum disorder–like symptoms: a comparative study. *International Journal of Developmental Disabilities 2022, 68*(5):744-755. | Ineligible outcomes | no targeted movement behaviors |
| Schlarb 2012 | Schlarb AA, Brandhorst I: Mini-Kiss online: An Internet-based intervention program for parents of young children with sleep problems - Influence on parental behavior and children's sleep. *Nature and Science of Sleep 2012, 4*:41-52. | Ineligible study design | not RCT |
| Taveras 2012 | Taveras, E. M., McDonald, J., O'Brien, A., Haines, J., Sherry, B., Bottino, C. J., Troncoso, K., Schmidt, M. E., & Koziol, R. (2012). Healthy Habits, Happy Homes: Methods and baseline data of a randomized controlled trial to improve household routines for obesity prevention. *Preventive Medicine, 55*(5), 418-426. | Ineligible outcomes | baseline data |
| Thorén 2020 | Thorén A, Janson A, Englund E, Silfverdal SA: Development, implementation and early results of a 12-week web-based intervention targeting 51 children age 5–13 years and their families. *Obesity Science and Practice 2020, 6*(5):516-523. | Ineligible participants | children aged 5–13 years |
| Tomayko 2021 | Tomayko EJ, Webber EJ, Cronin KA, Prince RJ, Adams AK: Use of Text Messaging and Facebook Groups to Support the Healthy Children, Strong Families 2 Healthy Lifestyle Intervention for American Indian Families. *Current Developments in Nutrition 2021, 5*:32-39. | Ineligible outcomes | no targeted movement behaviors |
| Trost 2014 | Trost SG, Sundal D, Foster GD, Lent MR, Vojta D: Effects of a pediatricweight management program with and without active video games a randomized trial. *JAMA Pediatrics 2014, 168*(5):407-413. | Ineligible participants | Mean (SD) age of the participants was 10.0 (1.7) years |
| Vann 2013 | Vann LH, Stanford FC, Durkin MW, Hanna A, Knight LM, Stallworth JR: "Moving and losing": A pilot study incorporating physical activity to decrease obesity in the pediatric population. *Journal of the South Carolina Medical Association (1975) 2013, 109*(4):116-120. | Ineligible participants | children and adolescents aged 5 to 18 years |
| Ward 2020 | Ward S, Belanger M, Leis A: Comparison between the Healthy Start-Depart Sante online and in-person training of childcare educators to improve healthy eating and physical activity practices and knowledge of physical activity and fundamental movement skills: A controlled trial. *Preventive Medicine Reports 2020, 20*:101264. | Ineligible outcomes | educators' PA knowledge |
| Willis 2016 | Willis TA, Roberts KPJ, Berry TM, Bryant M, Rudolf MCJ: The impact of HENRY on parenting and family lifestyle: A national service evaluation of a preschool obesity prevention programme. *Public Health 2016, 136*:101-108. | Ineligible study design | pre-post (not RCT) |
| Wingo 2020 | Wingo BC, Yang D, Davis D, Padalabalanarayanan S, Hopson B, Thirumalai M, Rimmer JH: Lessons learned from a blended telephone/e-health platform for caregivers in promoting physical activity and nutrition in children with a mobility disability. *Disability and Health Journal 2020, 13*(1). | Ineligible participants | Age of the children ranged from 6 to 17 years old. |
| Ng 2022 | Ng, M., Wenden, E., Lester, L., Westgarth, C., & Christian, H. (2022). A mobile health intervention to encourage physical activity in children: a randomised controlled trial. *BMC Pediatrics, 22*(1), 276. https://doi.org/10.1186/s12887-022-03336-9 | Ineligible participants | Children aged 5-10 years |
| Gerards 2015 | Gerards, S. M. P. L., Dagnelie, P. C., Gubbels, J. S., van Buuren, S., Hamers, F. J. M., Jansen, M. W. J., van der Goot, O. H. M., de Vries, N. K., Sanders, M. R., & Kremers, S. P. J. (2015). The Effectiveness of Lifestyle Triple P in the Netherlands: A Randomized Controlled Trial. *PLOS ONE, 10*(4), e0122240. https://doi.org/10.1371/journal.pone.0122240 | Ineligible participants | children 4–8 years old, overweight or obese |
| Staiano 2022 | Staiano, A. E., Newton, R. L., Jr., Beyl, R. A., Kracht, C. L., Hendrick, C. A., Viverito, M., & Webster, E. K. (2022). mHealth Intervention for Motor Skills: A Randomized Controlled Trial. *Pediatrics, 149*(5). https://doi.org/10.1542/peds.2021-053362 | Ineligible comparator | Both the control and intervention groups use eHealth. |
| Cloutier 2015 | Cloutier, M. M., Wiley, J., Huedo-Medina, T., Ohannessian, C. M., Grant, A., Hernandez, D., & Gorin, A. A. (2015). Outcomes from a Pediatric Primary Care Weight Management Program: Steps to Growing Up Healthy. *The Journal of Pediatrics*, *167*(2), 372-377.e371. https://doi.org/https://doi.org/10.1016/j.jpeds.2015.05.028 | Ineligible Study design | pre-post (not RCT) |
| Gao 2019 | Gao, Z., Zeng, N., Pope, Z. C., Wang, R., & Yu, F. (2019). Effects of exergaming on motor skill competence, perceived competence, and physical activity in preschool children. *Journal of Sport and Health Science, 8*(2), 106-113. | Ineligible Study design | 2-arm experimental design with repeated measures |
| Corkum 2019 | Corkum, P., Chambers, C., Godbout, R., Gruber, R., Hall, W., Reid, G., Stremler, R., Weiss, S., Witmans, M., Rigney, G., Begum, E., & Andreou, P. (2019). Results of an 8-month pan-Canadian randomized controlled trial of an internet-based behavioral intervention for pediatric insomnia, the better nights, better days program. *Sleep Medicine, 64*(Supplement 1), S80. | No full text | Not a full-length study report (e.g. conference abstract) |
| Wald 2018 | Wald, E. R., Ewing, L. J., Moyer, S. C. L., & Eickhoff, J. C. (2018). An Interactive Web-Based Intervention to Achieve Healthy Weight in Young Children. *Clinical Pediatrics, 57*(5), 547-557. https://doi.org/10.1177/0009922817733703 | Ineligible participants | chidren aged 3-7 |
| Taveras 2011 | Taveras, E. M., Gortmaker, S. L., Hohman, K. H., Horan, C. M., Kleinman, K. P., Mitchell, K., Price, S., Prosser, L. A., Rifas-Shiman, S. L., & Gillman, M. W. (2011). Randomized controlled trial to improve primary care to prevent and manage childhood obesity: the High Five for Kids study. *Arch Pediatr Adolesc Med, 165*(8), 714-722. https://doi.org/10.1001/archpediatrics.2011.44 | Ineligible intervention | intervention modes: motivational interviewing by clinicians and educational modules (lack of eHealth intervention) |
| Rifas-Shiman 2017 | Rifas-Shiman, S. L., Taveras, E. M., Gortmaker, S. L., Hohman, K. H., Horan, C. M., Kleinman, K. P., Mitchell, K., Price, S., Prosser, L. A., & Gillman, M. W. (2017). Two-year follow-up of a primary care-based intervention to prevent and manage childhood obesity: the High Five for Kids study. *Pediatr Obes, 12*(3), e24-e27. https://doi.org/10.1111/ijpo.12141 | Ineligible intervention | intervention modes: motivational interviewing by clinicians and educational modules (lack of eHealth intervention) |
