# Supplementary material for: Effects of eHealth Interventions on 24-Hour Movement Behaviors Among Preschoolers: Systematic Review and Meta-Analysis
Source: J Med Internet Res. 2024 Feb 21;26:e52905. doi: 10.2196/52905 (PMC10918543; doi:10.2196/52905)
Supplement: Multimedia Appendix 7 [file jmir_v26i1e52905_app7.docx]

**Supplementary Material 5: Number of included studies per country and income economy**

(according to The World Bank classification)

| **Country*** | **Number of studies** | **Income economy** |
| --- | --- | --- |
| Australia | 3 | High |
| New Zealand | 1 | High |
| Norway | 1 | High |
| Sweden | 3 | High |
| Turkey | 1 | Upper-middle |
| USA | 10 | High |

*Countries are listed in an alphabetical order

Source: The World Bank. World Bank Country and Lending Groups – Country Classification. Available from <https://datahelpdesk.worldbank.org/knowledgebase/articles/906519-world-bank-country-and-lending-groups>. Accessed on April 9, 2023.
