# Supplementary material for: Effects of eHealth Interventions on 24-Hour Movement Behaviors Among Preschoolers: Systematic Review and Meta-Analysis
Source: J Med Internet Res. 2024 Feb 21;26:e52905. doi: 10.2196/52905 (PMC10918543; doi:10.2196/52905)
Supplement: Multimedia Appendix 10 [file jmir_v26i1e52905_app10.docx]

**Meta-analysis**

Forest plots of moderate to vigorous physical activity and sedentary behaviors in MINISTOP project


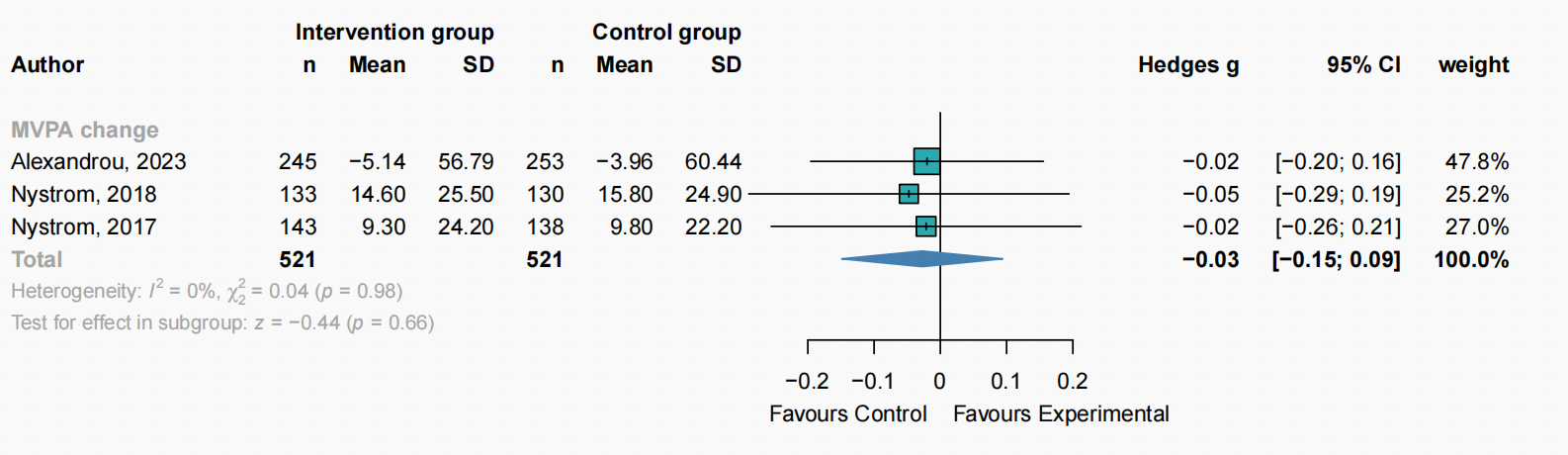


Figure s1. Forest plot of the effects of eHealth intervention on MVPA change.


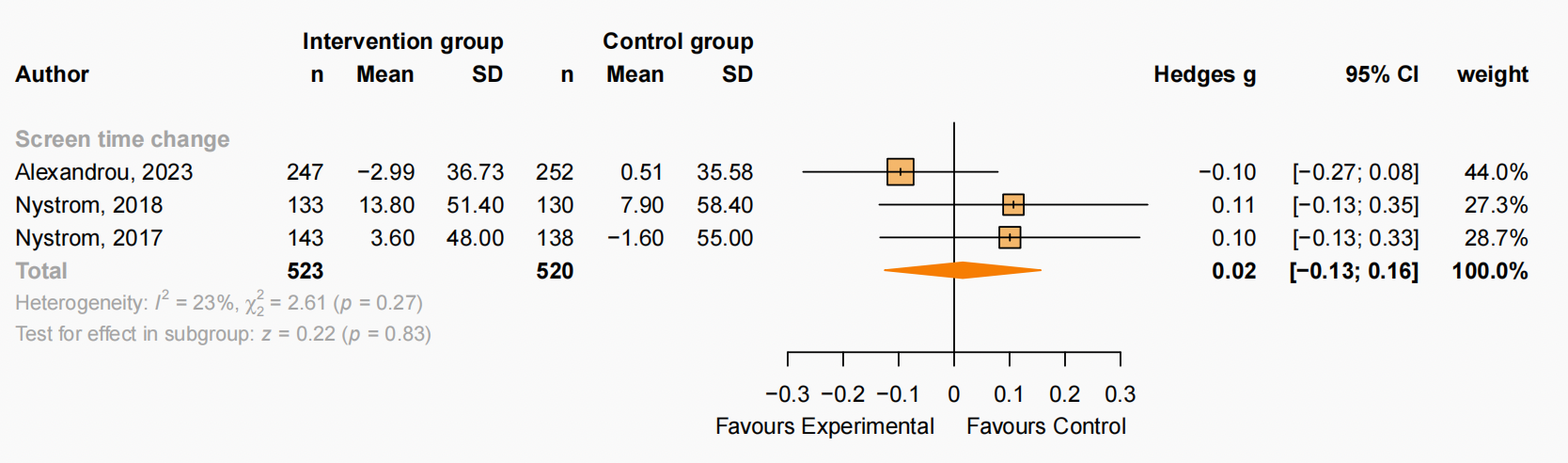


Figure s2. Forest plot of the effects of eHealth intervention on sedentary behaviour change.
