# Supplementary material for: Effects of eHealth Interventions on 24-Hour Movement Behaviors Among Preschoolers: Systematic Review and Meta-Analysis
Source: J Med Internet Res. 2024 Feb 21;26:e52905. doi: 10.2196/52905 (PMC10918543; doi:10.2196/52905)
Supplement: Multimedia Appendix 11 [file jmir_v26i1e52905_app11.docx]

**Subgroup analysis**

Forest plots of the subgroup analyses of moderate to vigorous physical activity and sedentary behaviors


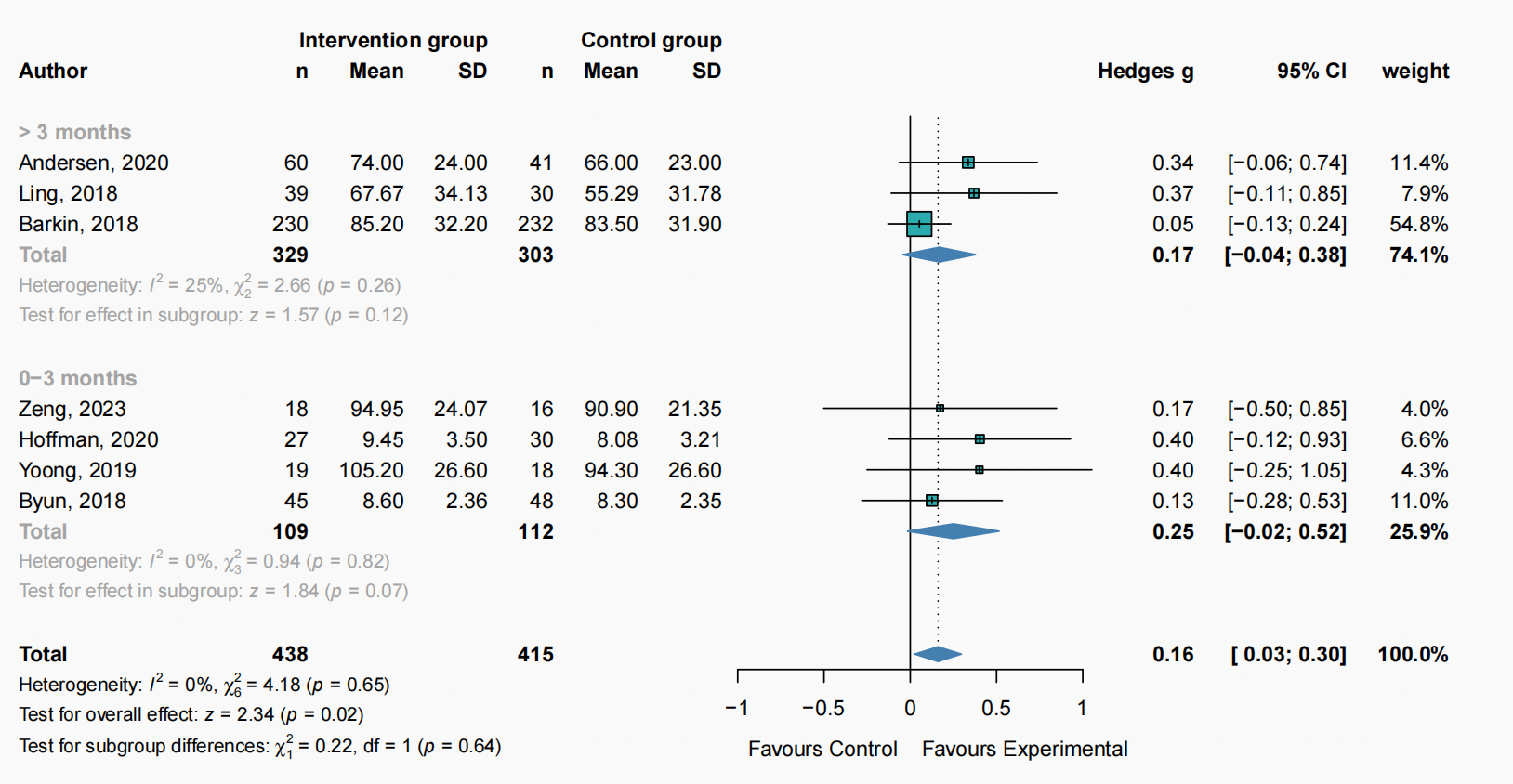


Figure S1. Forest plot of the subgroup analysis of the effects of eHealth intervention on MVPA (Intervention duration: 0-3 months or > 3 months).


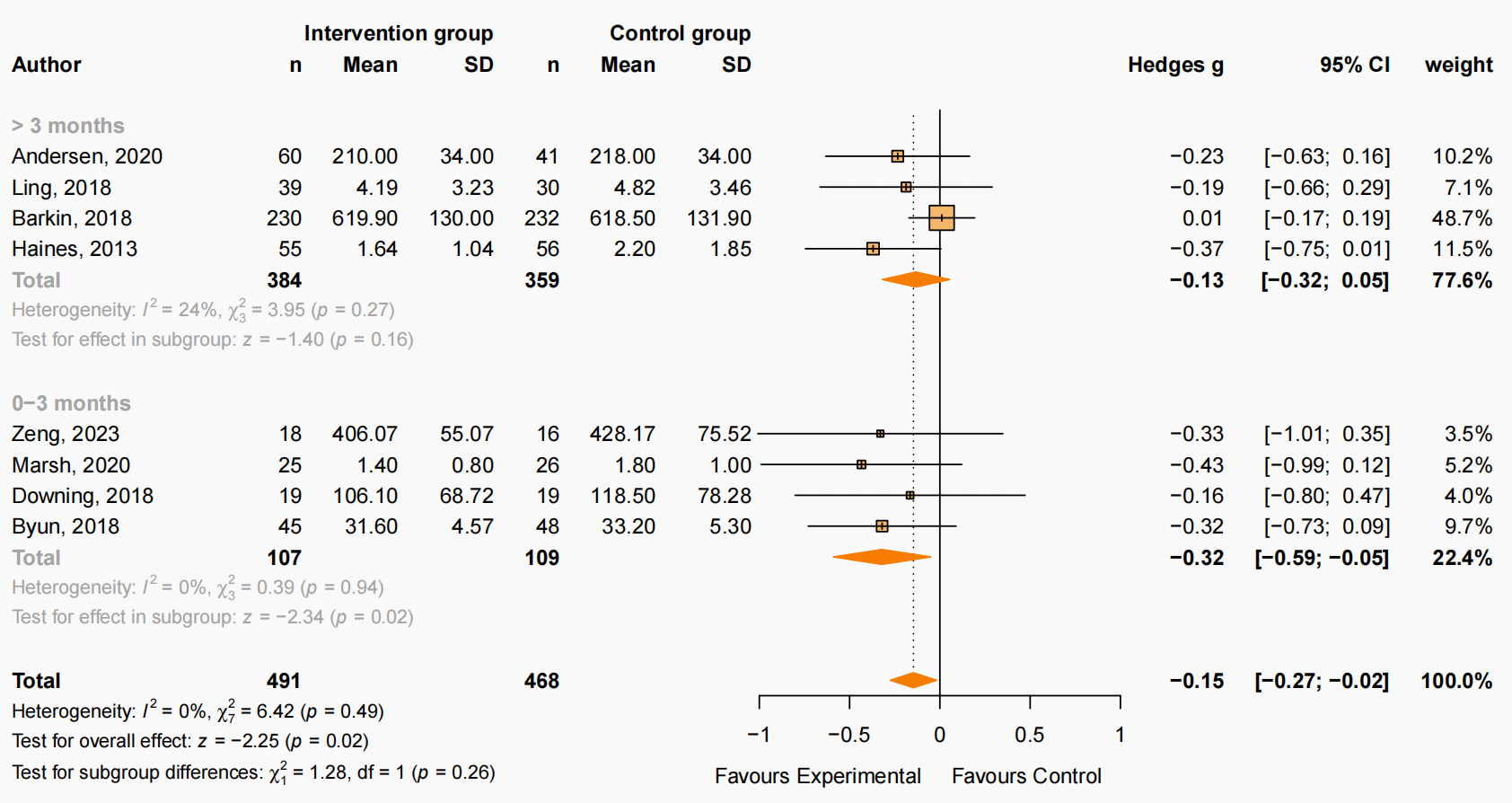


Figure S2. Forest plot of the subgroup analysis of the effects of eHealth intervention on sedentary behaviour (Intervention duration: 0-3 months or > 3 months).


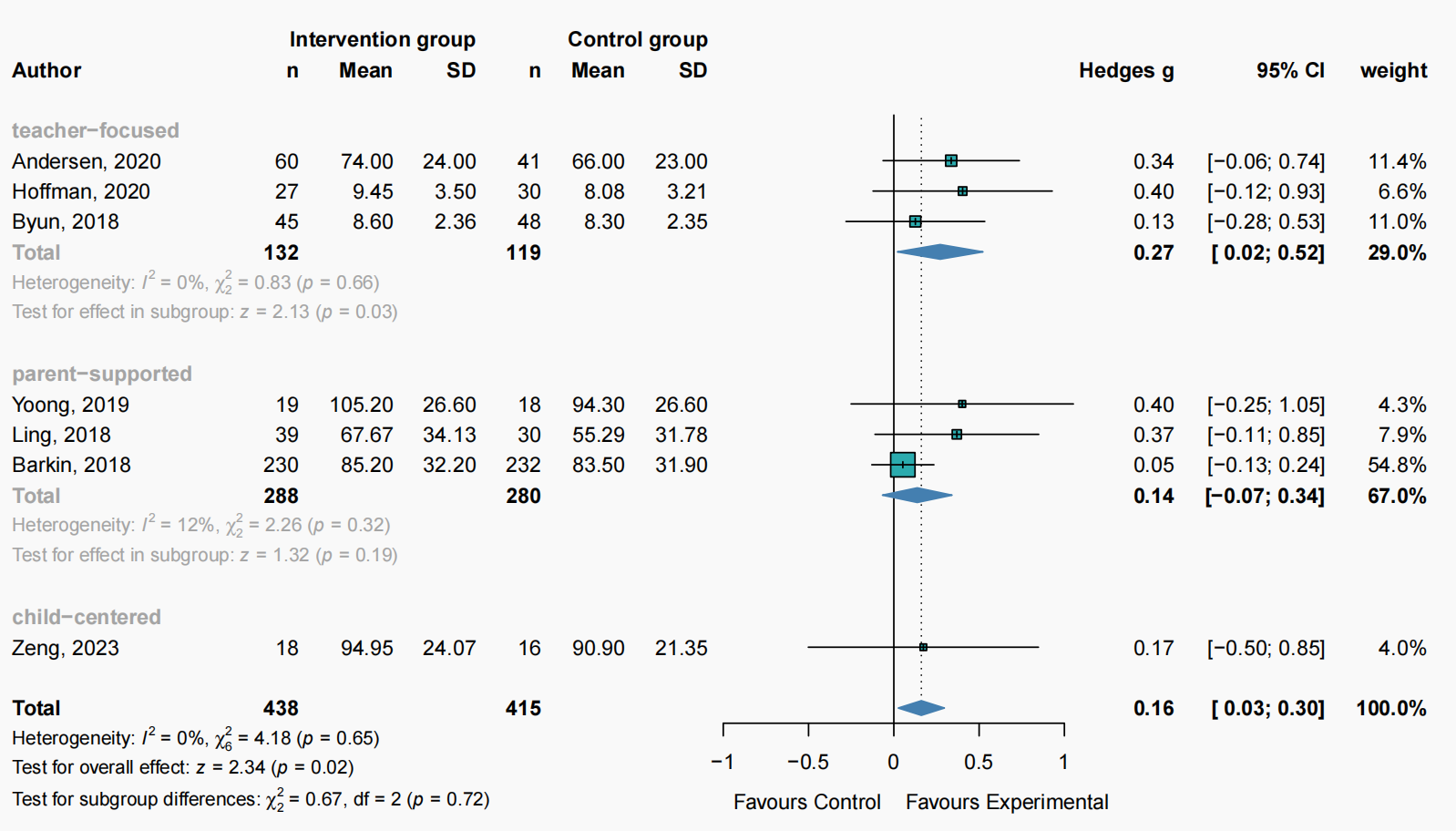


Figure S3. Forest plot of the subgroup analysis of the effects of eHealth intervention on MVPA (Intervention type: teacher-led, parents-focused or child-centered).


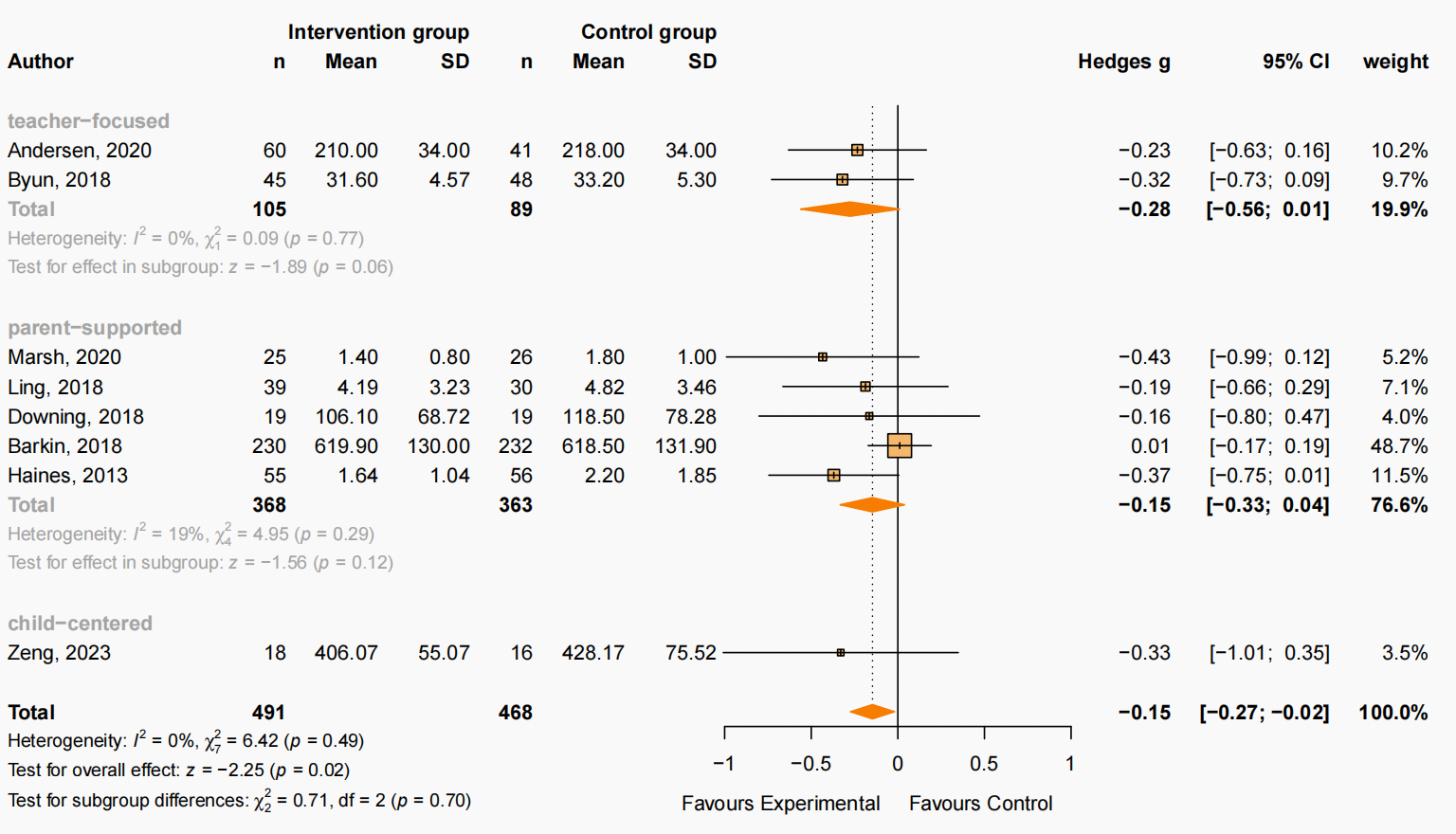


Figure S4. Forest plot of the subgroup analysis of the effects of eHealth intervention on sedentary behaviour (Intervention type: teacher-led, parents-focused or child-centered).


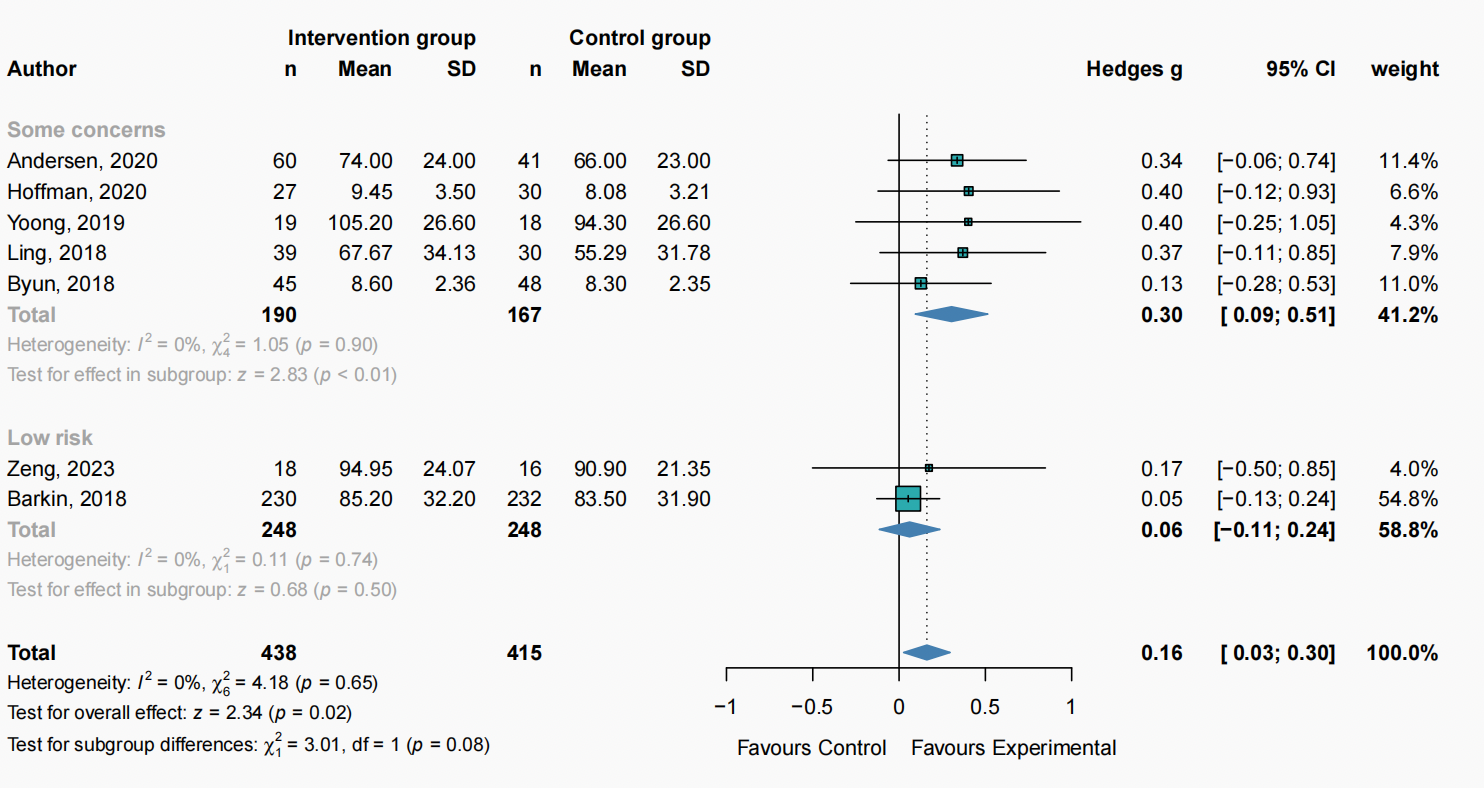


Figure S5. Forest plot of the subgroup analysis of the effects of eHealth intervention on MVPA (Risk of bias: some concerns or low risk).


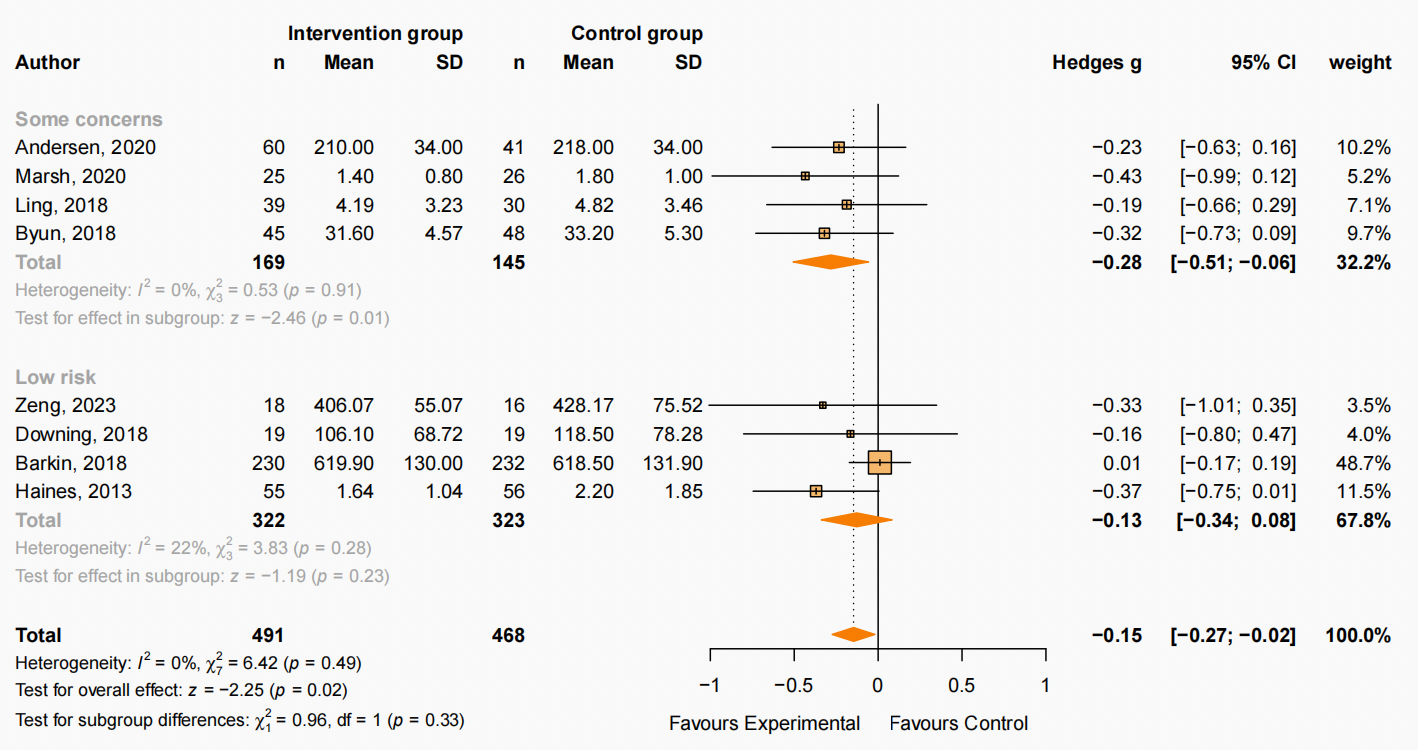


Figure S6. Forest plot of the subgroup analysis of the effects of eHealth intervention on sedentary behaviour (Risk of bias: some concerns or low risk).


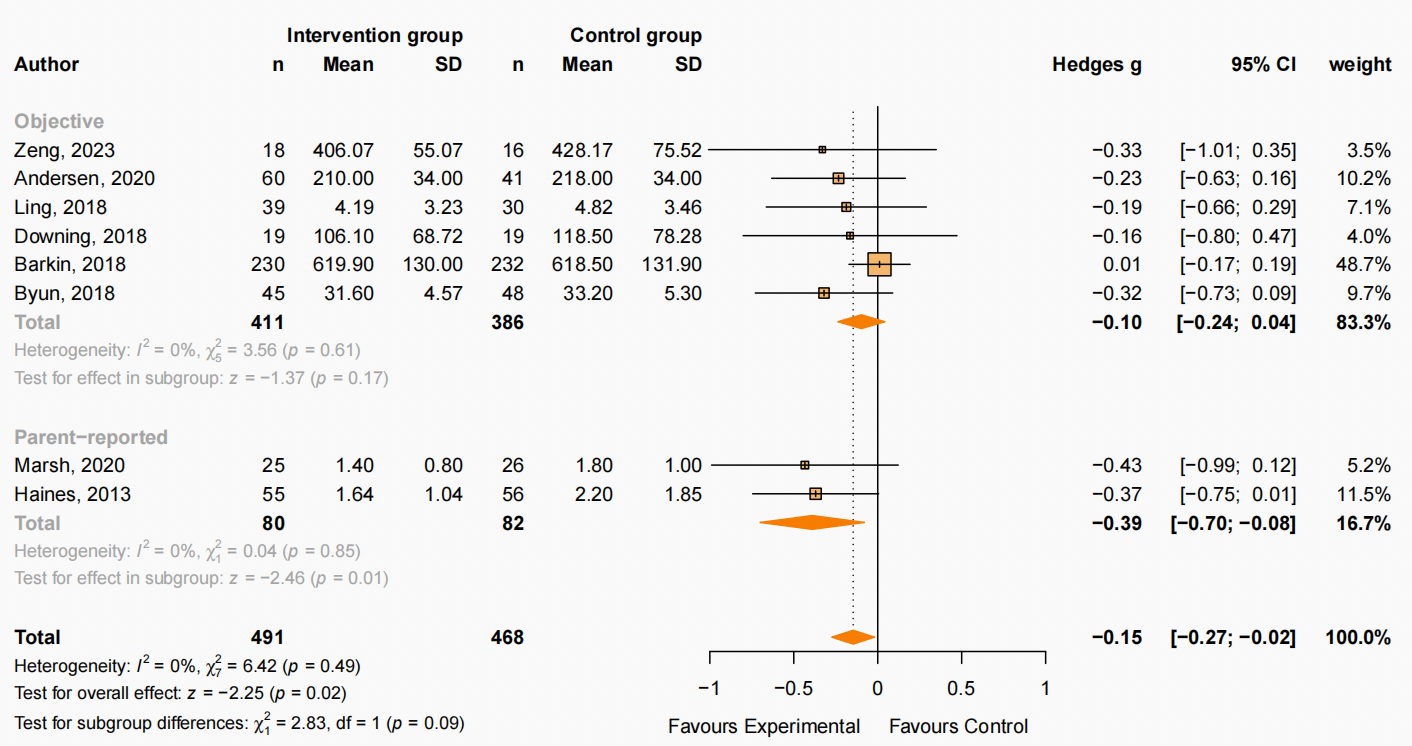


Figure S7. Forest plot of the subgroup analysis of the effects of eHealth intervention on sedentary behaviour (Measurement tool: self- reported or objective).

***RESULTS OF SUBGROUP ANALYSES***

*MVPA*

Results of the subgroup analyses identified not significantly different changed in MVPA by intervention duration (0-3 months vs. >3 months, [*g* = 0.25, 95% CI: -0.02; 0.52 vs. *g* = 0.17, 95% CI: -0.04; 0.38, *p* = 0.67; Figure S1]). In figure S3, the results revealed a significantly increase on MVPA than in the control group in case of the child-centered group (*g* = 0.17, 95% CI: -0.50; 0.85) and teacher-led group (*g* = 0.27, 95% CI: 0.02; 0.52, *p* = 0.03). In the case of parent-focused group, no significant difference between groups were observed (*g* = 0.14, 95% CI: -0.07; 0.34, *p* = 0.19). For risk of bias, studies in some concerns demonstrated higher MVPA, compared to control group (*g* = 0.30, 95% CI: 0.09; 0.51, *p* < 0.08; Figure S5). However, low risk studies did not achieve a statistically significant MVPA increasing (*g* = 0.06, 95% CI: -0.11; 0.24, *p* = 0.50; Figure S5).

*Sedentary behaviors*

In figure S2, interventions with an estimated duration of 0-3 months (*g* = -0.32, 95% CI: -0.59; -0.05; *p* = 0.02; I^2^=0%) had a higher intervention effect size on Sedentary behaviors than those with an intervention duration >3 months (*g* = -0.13, 95% CI: -0.32; 0.05; *p* = 0.16; I^2^=0%), but it did not present statistically significant decrease. The results of the subgroup analyses indicated that sedentary behaviors had not changed significantly by intervention type (child−centered, teacher-focused, parent-supported, [*g* = -0.33, 95% CI: -1.01; 0.35 vs. *g* = -0.28, 95% CI: -0.56; 0.01 vs. *g* = -0.15, 95% CI: −0.33; 0.04, *p* = 0.70; Figure S4]), risk of bias (some concerns vs. low risk, [*g* = -0.28, 95% CI: -0.51; -0.06 vs. *g* = -0.13, 95% CI: −0.34; 0.08, *p* = 0.33; Figure S6]), measurement method (objective vs. self-report, [*g* = -0.1, 95% CI: -0.24; 0.04 vs. *g* = -0.39, 95% CI: -0.70; -0.08, *p* = 0.09; Figure S7]).
