# Supplementary figures and images for: Effects of eHealth Interventions on 24-Hour Movement Behaviors Among Preschoolers: Systematic Review and Meta-Analysis
Source: J Med Internet Res. 2024 Feb 21;26:e52905. doi: 10.2196/52905 (PMC10918543; doi:10.2196/52905)

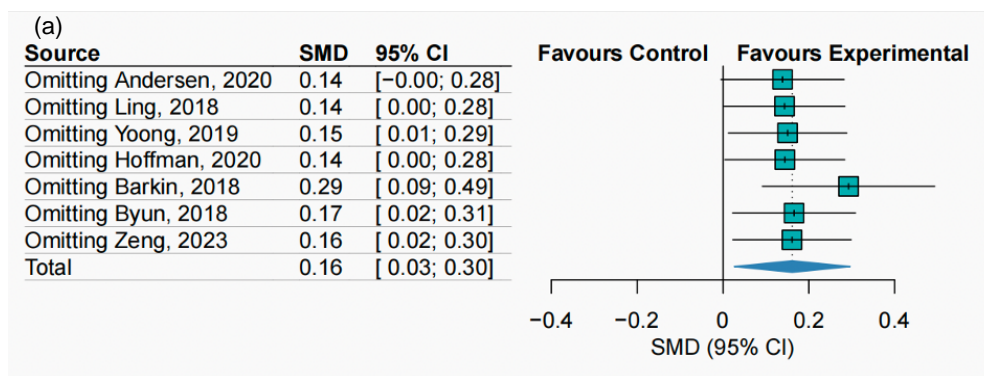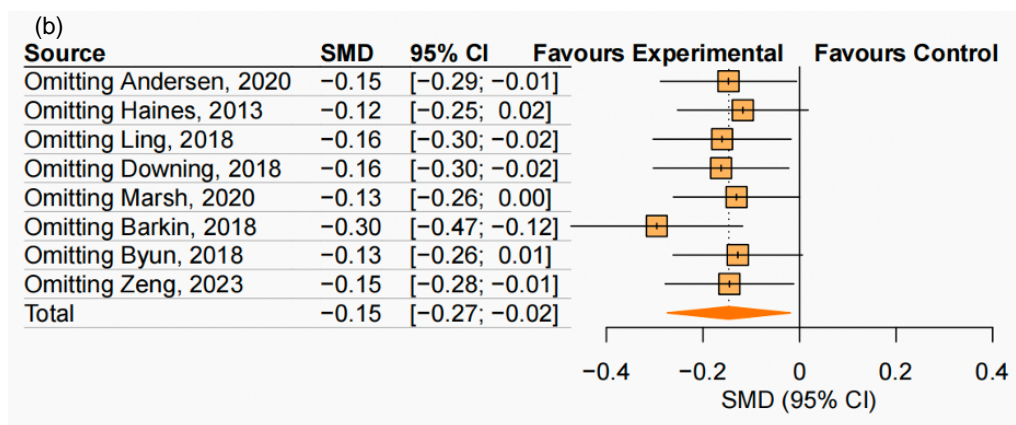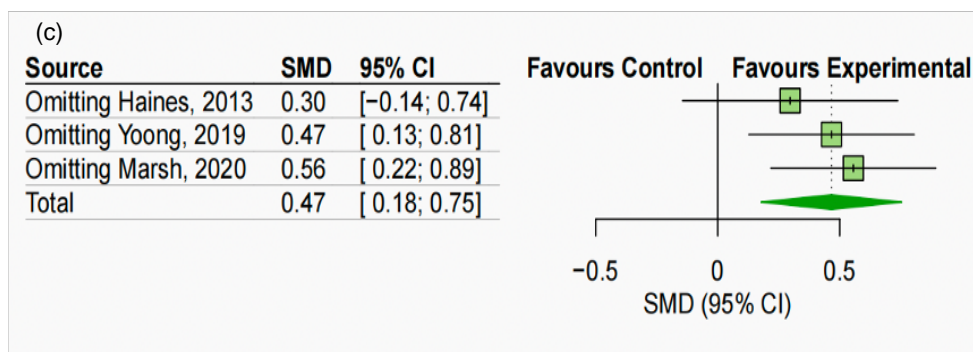

Fig S10. Forest plot: sensitivity analyses for (a) MVPA; (b) SED; (c) sleep

Supplement: Multimedia Appendix 12 [file jmir_v26i1e52905_app12.pdf]

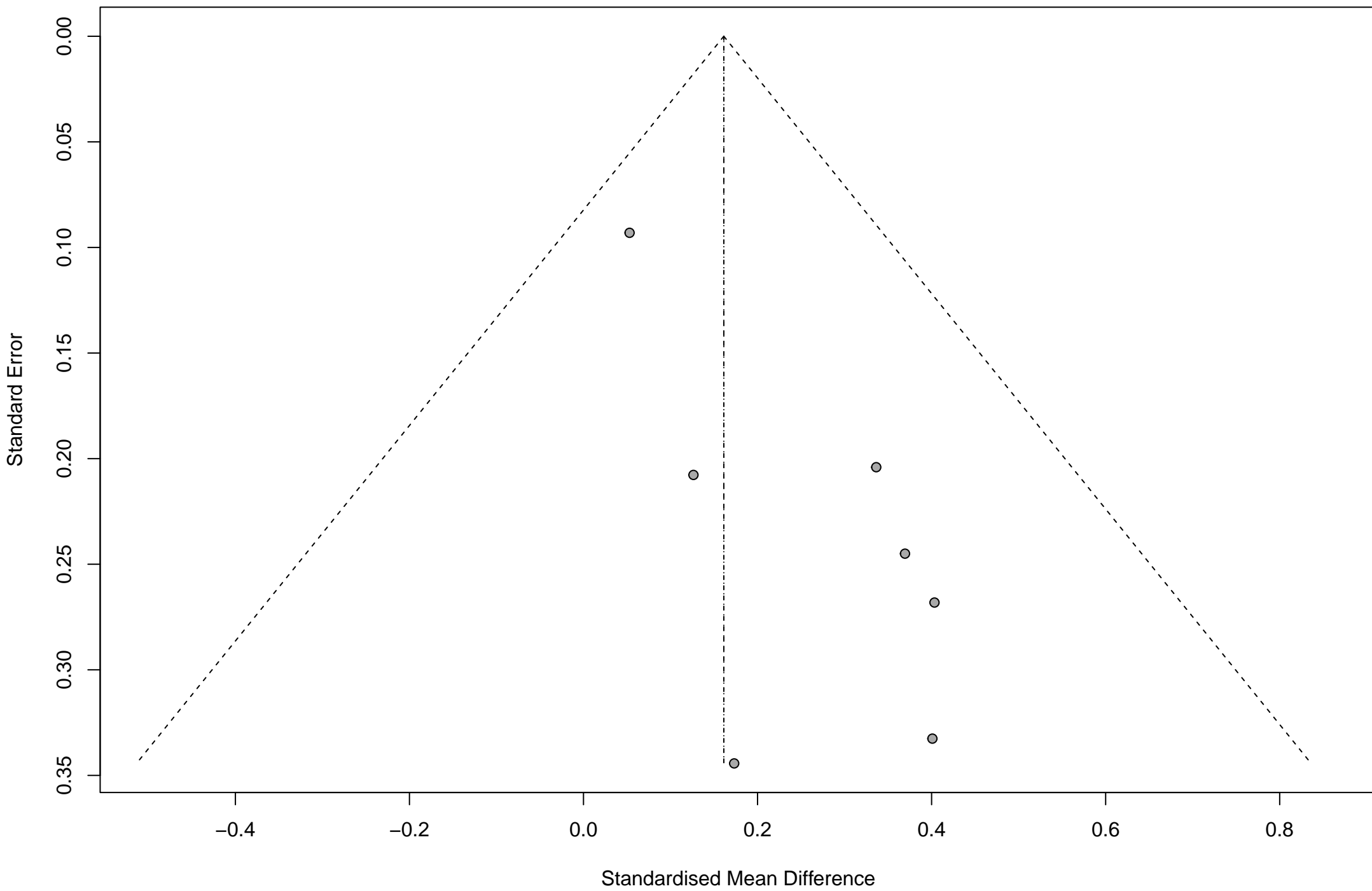

Supplement: Multimedia Appendix 13 [file jmir_v26i1e52905_app13.pdf]

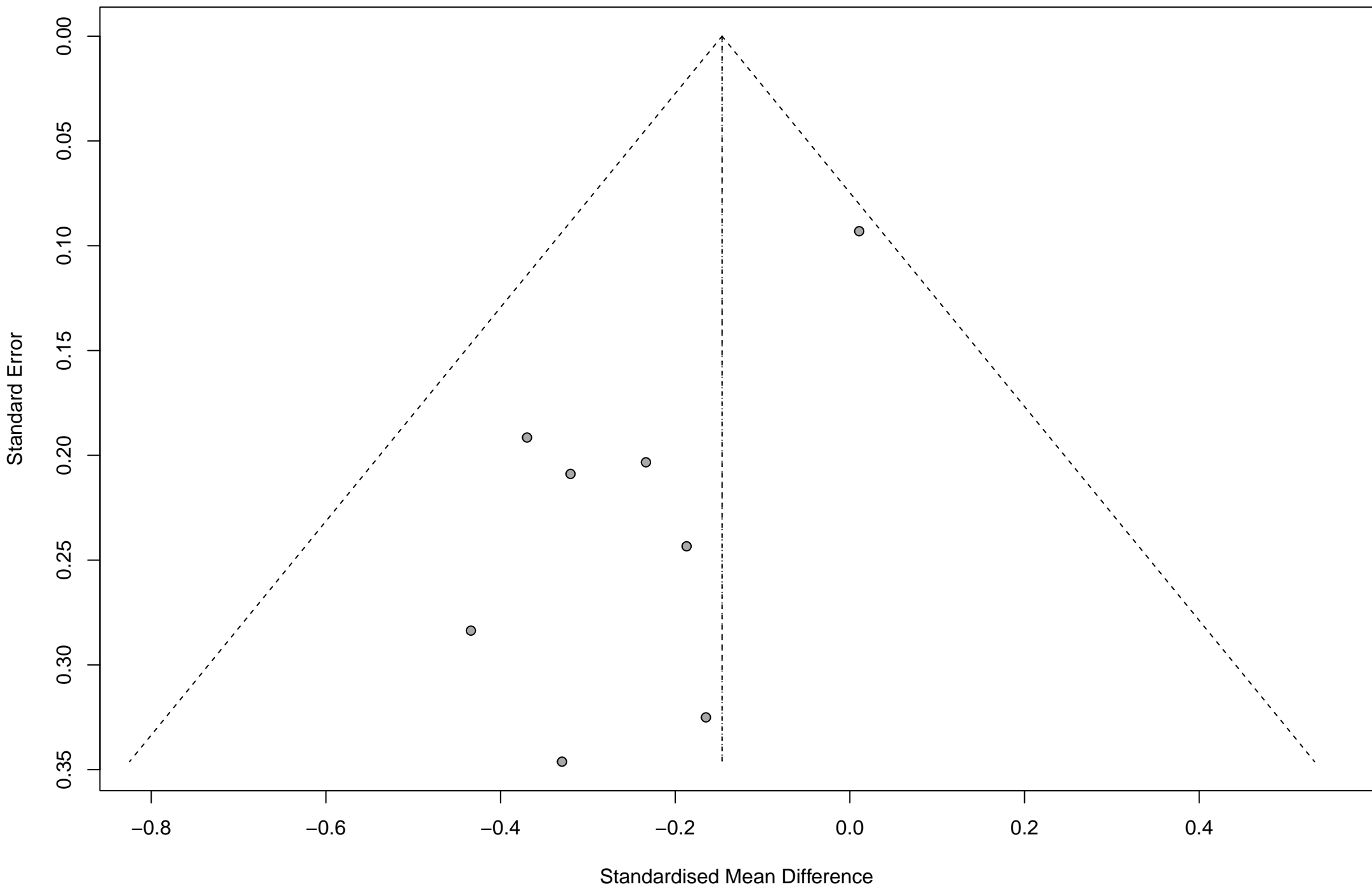

Supplement: Multimedia Appendix 14 [file jmir_v26i1e52905_app14.pdf]
