# Supplementary material for: Effects of eHealth Interventions on 24-Hour Movement Behaviors Among Preschoolers: Systematic Review and Meta-Analysis
Source: J Med Internet Res. 2024 Feb 21;26:e52905. doi: 10.2196/52905 (PMC10918543; doi:10.2196/52905)
Supplement: Multimedia Appendix 15 [file jmir_v26i1e52905_app15.pdf]

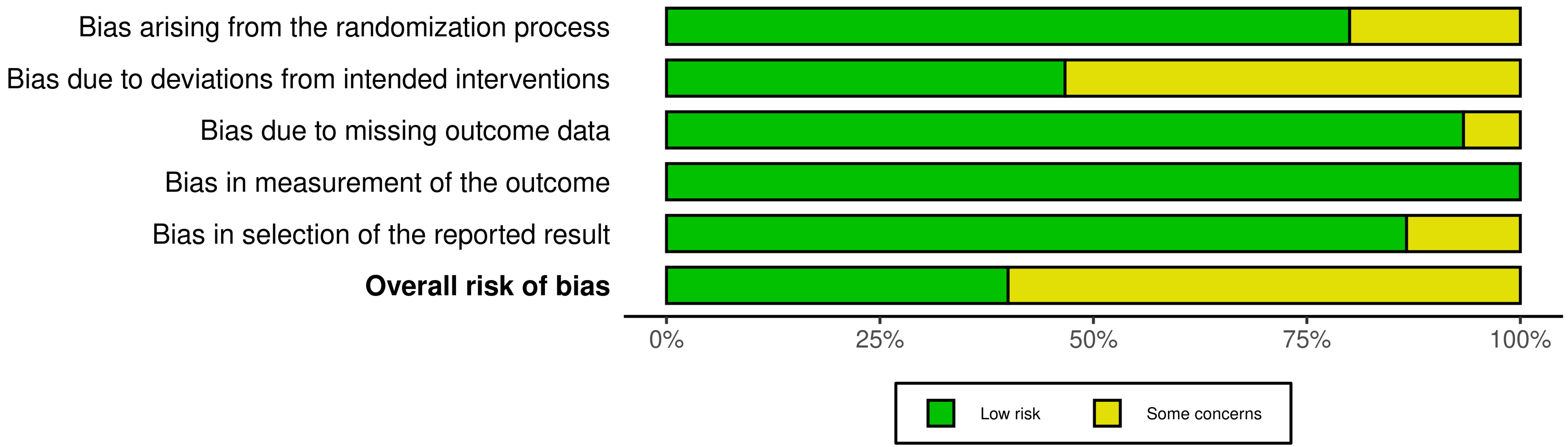
Bias arising from the randomization process
Bias due to deviations from intended interventions
Bias due to missing outcome data
Bias in measurement of the outcome
Bias in selection of the reported result
Overall risk of bias
0%
25%
50%
75%
100%
Low risk
Some concerns

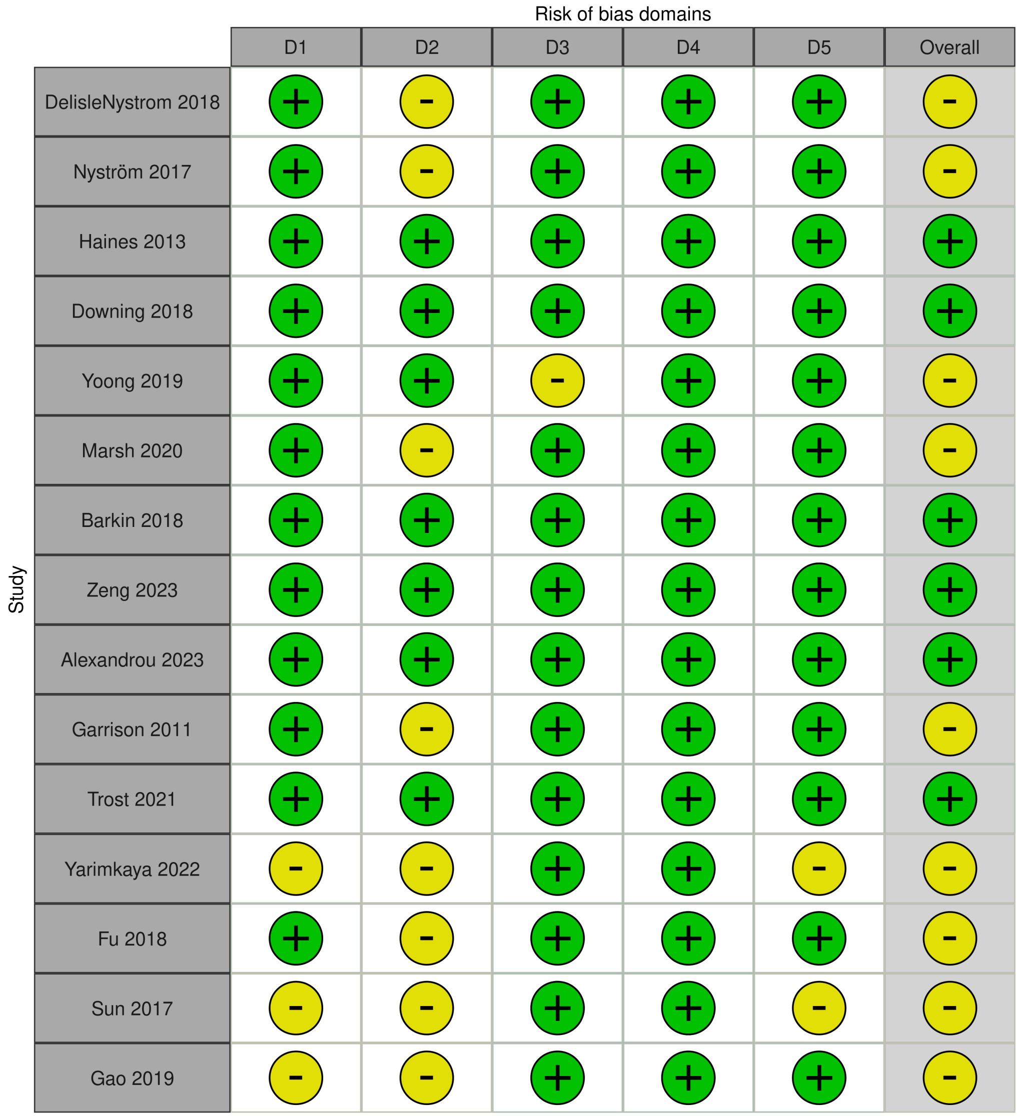

Domains:
D1: Bias arising from the randomization process.
D2: Bias due to deviations from intended intervention.
D3: Bias due to missing outcome data.
D4: Bias in measurement of the outcome.
D5: Bias in selection of the reported result.

Judgement
- Some concerns
+ Low

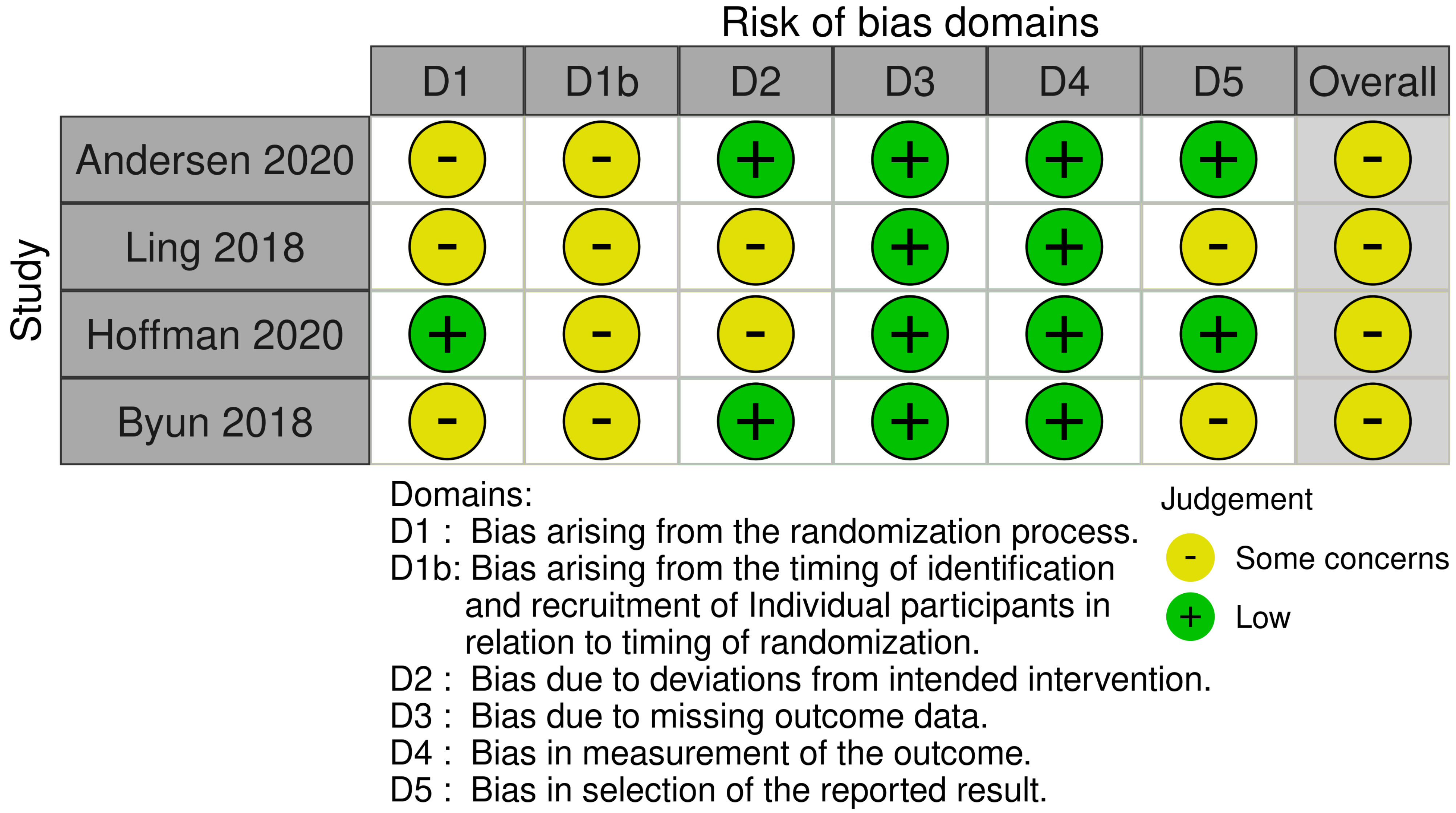
Risk of bias domains
D1
D1b
D2
D3
D4
D5
Overall
Study
Andersen 2020
Ling 2018
Hoffman 2020
Byun 2018
Domains:
D1 : Bias arising from the randomization process.
D1b: Bias arising from the timing of identification and recruitment of Individual participants in relation to timing of randomization.
D2 : Bias due to deviations from intended intervention.
D3 : Bias due to missing outcome data.
D4 : Bias in measurement of the outcome.
D5 : Bias in selection of the reported result.
Judgement
Some concerns
Low
